# Implementation of two transdiagnostic interventions based on emotional regulation for professionals who treat alcohol addiction in the Spanish mental health system: A multicenter mixed methods pilot study protocol

**DOI:** 10.1371/journal.pone.0318512

**Published:** 2025-05-09

**Authors:** María V. Navarro-Haro, Alba Abanades-Morillo, Óscar Péris-Baquero, Verónica Martínez-Borba, Elena Crespo-Delgado, Abel Baquero-Escribano, Laura Masferrer-Boix, Jorge Osma

**Affiliations:** 1 Department of Psychology and Sociology, Faculty of Social and Human Sciences, University of Zaragoza, Teruel, Spain; 2 Department of Basic Psychology, Clinical Psychology and Psychobiology, University Jaume I, Castellón de la Plana, Spain; 3 Department of Medicine and Surgery, Faculty of Health Sciences, University CEU Cardenal Herrera, Valencia, Spain; 4 Department of Psychology, Faculty of Education and Psychology, University of Girona, Girona, Spain; PLOS: Public Library of Science, UNITED KINGDOM OF GREAT BRITAIN AND NORTHERN IRELAND

## Abstract

Emotional dysregulation has been considered a transdiagnostic factor and an important determinant of craving and relapse in alcohol addiction. Dialectical Behavior Therapy (DBT) and the Unified Protocol (UP) are two transdiagnostic emotional regulation programs with good efficacy in improving alcohol addiction severity. However, an important barrier encountered when implementing evidence-based interventions in drug addiction services is the inadequate training received by professionals. This study aims to evaluate the effect of a dissemination and pilot implementation initiative of DBT and UP among professionals treating alcohol addiction in the national Spanish healthcare system. Methods: The study will be conducted in two phases using a mixed methods design. In phase 1, two 3-day training workshops (DBT and UP; 40 hours total) will be provided by experts to at least 130 healthcare workers from three Spanish regions. Participants will be randomly assigned to receive either DBT or UP training first, followed by the other. The study will include a range of quantitative outcomes including beliefs about alcohol abuse, burnout, self-efficacy, attitudes towards evidence-based interventions, organizational variables, acceptability and intention to use the interventions, barriers to implementation, and knowledge acquisition. The appropriateness of the interventions in real community settings will be qualitatively assessed. In phase 2, at least 30 trained professionals will be randomly assigned to implement DBT or/and UP 3-month group interventions in their workplaces with alcohol addiction patients. Quantitative outcomes will include acceptability, feasibility, appropriateness, fidelity and sustainability of the interventions, barriers to implementation, as well as qualitative descriptions of barriers and facilitators during the implementation process. Discussion: To our knowledge, this is the first study to evaluate a pilot implementation of transdiagnostic psychological interventions based on emotion regulation to treat alcohol addiction. Findings of this study will inform of factors influencing the successful implementation of DBT and UP in community-based addiction services.

**Trial registration:** ClinicalTrials.gov NCT06366100.

## Introduction

### Background

#### Alcohol addiction and emotion dysregulation.

Alcohol use disorders (as defined by the American Psychological Association, APA) [1], including harmful drinking, are fairly common mental health disorders associated with significant morbidity and mortality [2,3]. A causal relationship between harmful alcohol consumption and several mental has been found [4]. Alcohol dependence is among the most frequent mental health problems in Spain, affecting more than 4% of the population [5], especially in outpatient drug addiction units of the Spanish Health System (e.g., about 44% of patients in Catalonia) [6].

People with alcohol addiction often suffer from a variety of adverse effects such as a pronounced reduction in quality of life and widespread psychosocial consequences such as violence, neglect, abuse, and absenteeism in the workplace, among others, resulting in a high social and economic burden [7]. For these reasons, improving the effectiveness of intervention programs to address alcohol addiction has generated great research interest [8].

The scientific literature suggests alcohol use as a dysfunctional emotion regulation (ER) strategy [9]. Some studies have revealed that mood and negative valence emotions are one of the most important determinants of craving and relapse in alcohol addiction [9,10]. ER difficulties have been also found to predict severity of alcohol use disorder [11,12,13]. People with alcohol addiction have shown greater use of maladaptive ER strategies [14] and presented higher neuroticism [15] and negative emotionality [16], compared to non-alcohol addiction controls. Therefore, addressing emotion dysregulation could represent a promising target for the improvement of psychological treatments for alcohol addiction.

#### Transdiagnostic interventions based on ER to treat alcohol addiction.

People with alcohol addiction often suffer from other comorbid disorders (such as personality disorders and emotional disorders; hereafter referred to as EDs: anxiety, depression and related disorders, [17]) that complicate treatment and negatively influence treatment outcomes [8]). In the last decade, new transdiagnostic treatment proposals have emerged that aim to address mechanisms shared by different disorders [18].

Some of these interventions focus on ER as a key component of treatment, allowing different comorbidities to be addressed at the same time [19]. Evidence supports the notion of emotional dysregulation as a transdiagnostic construct [20–23] across EDs [24, 25,26]. A recent meta-analysis on the effectiveness of ER interventions in individuals with substance dependence found moderate and significant effect sizes in reducing substance use (including alcohol) and increasing ER across psychological and physiological measures [27]. Dialectical Behavior Therapy [DBT; 28,29] and the Unified Protocol for transdiagnostic treatment of EDs [UP, 30] are two examples of transdiagnostic psychological interventions to address emotional dysregulation in different psychological disorders with good results [31–34].

Regarding DBT, recent evidence has shown that the group skills training component of DBT alone is effective in reducing dysfunctional behaviors associated with emotional dysregulation for several clinical conditions [34], including alcohol addiction [12,35]. DBT has been adapted to treat Substance Use Disorders [DBT-SUD; 35] incorporating concepts and modalities designed to promote abstinence and reduce the impact of substance relapse [35]. Studies in which patients with dual diagnosis were treated with DBT-SUD found significant reductions in heavy drinking and emotional dysregulation from pre-intervention to post-intervention and 6-month follow-up [36,37] and significant decreases in the severity and substance use [38]. Other studies conducted in Italy observed low rates of alcohol relapse and treatment dropout after a 3-month group DBT skills training program [39]. Those studies also showed preliminary support for reductions in addiction severity, consecutive days of abstinence and emotional dysregulation, which in turn mediated changes in secondary measures such as experiential avoidance [13,40].

Concerning UP, there are already three systematic review and meta-analysis studies reporting the effectiveness of UP in improving the use of adaptive strategies in eating disorders [31–33], as well as others with more severe difficulties in ER such as bipolar disorder [41], people with self-injurious behaviors [42] or borderline personality disorder [43]. Regarding alcohol abuse, a study with a double-blind randomized placebo-controlled design showed efficacy of UP in reducing the percentage of heavy drinking days in a sample with comorbid anxiety disorders [42]. In addition, adaptations and recommendations of the UP in a group format for comorbid EDs and substance use disorders have been published [44].

#### Dissemination and Implementation of evidence-based psychological treatments for alcohol addiction.

Evidence shows that translation of efficacy studies to clinical practice faces significant challenges, with an estimated time lag of 17–20 years from study to implementation [45,46]. Implementation science aims to address this problem by studying methods that promote the integration of research results into daily practice, thereby improving the quality and efficiency of healthcare services [47]. Dissemination and implementation research in the addictions field is relatively new [48], leading to limited knowledge about optimizing the implementation of evidence-based psychological treatments (EBPTs). A key barrier to the implementation of alcohol addiction interventions is the need for adequate training in EBPTs for professionals. Research also suggests that professionals’ familiarity with the interventions, perceived efficacy and attitudes toward EBPTs are associated with successful implementation [46,47,49].

DBT implementation research to address emotion dysregulation indicates high adoption rates of DBT treatment modes (individual therapy, group skills training, phone coaching and consultation team meetings) among mental health professionals worldwide after DBT Intensive Training [50–53], with organizational support suggested as a critical factor for successful implementation [50]. Barriers to DBT implementation include the lack of administrative commitment, funding issues, and insufficient resources. On the other hand, the provider’s familiarity with the intervention, positive attitudes toward EBPs and therapy, and a supportive organizational climate and openness to the implementation of new interventions are proposed as DBT implementation facilitators [54,55].

Regarding UP, a study conducted in United States [56] found that trained practitioners positively valued the flexibility and relative advantage of UP over standard EBPTs, but they also suggested that the barriers encountered in the implementation of other EBPTs (i.e., lack of administrative support or time for training) also appeared in the case of UP. Another study [57] with professionals working in a safety net for homeless people (including those with addiction) found good attitudes towards EBPs that were maintained after UP training, and high rates of fidelity in the application of UP with their patients. In Spain, UP studies have shown high acceptability and intention to use the UP among psychologists after receiving the training [58,59]. However, more research is needed to understand the specific factors that influence the implementation process among professionals trained in DBT or UP, especially when treating alcohol use disorder.

#### Implementation science in EBPTs.

The research methodology used in EBPTs dissemination and implementation trials differs from efficacy and effectiveness trials. Implementation studies test the intervention(s) in real-world settings using flexible protocols tailored to the specific needs of the service [60]. To frame the implementation strategies of an intervention, different models have been developed such as the PARHIS (Promoting Action on Research Implementation in Health Services; [61], the RE-AIM (Reach, Effectiveness, Adoption, Implementation and Maintenance of the intervention; [62]) and the Consolidated Framework for Implementation Research [CFIR; 63], being the former the most widely used.

Based on these models, Proctor et al. [64] proposed a series of key outcome variables to be evaluated in treatment implementation studies (e.g., acceptability, adoption, appropriateness, feasibility, fidelity, sustainability, etc.). In this study, we will evaluate the main outcome variables that have been suggested to test treatment implementation. Two sequential study phases (lasting one year each) to evaluate dissemination and implementation are proposed.

## Methods

### Aims

#### Phase 1: DBT and UP training for professionals treating alcohol addiction and pre-implementation.

The general objective of phase 1 is to assess the effect of DBT and UP training (two 3-day workshops) in mental health professionals treating people with alcohol addiction and to collect pre-implementation relevant information in order to adapt these interventions for clinical practice.

Specific aims:

1. To assess acceptability, intention to use, as well as appropriateness, feasibility of DBT and UP interventions by mental health professionals after each training.
2. To evaluate attitudes toward EBTs, level burnout and climate about implementing DBT and UP treatments of mental health professionals before receiving the training.
3. To assess beliefs about the nature of alcohol addiction of mental health professionals before and after DBT and UP training.
4. To explore barriers, facilitators and organizational readiness to implement DBT and UP after each training.
5. To determine the level of knowledge acquisition in DBT and UP and satisfaction with the workshop after each training.
6. To collect the opinions and recommendations of mental health professionals working with people with alcohol addiction on the adaptations that should be made to facilitate implementation of the interventions (DBT and UP) in clinical practice.

#### Phase 2: Pilot implementation of DBT and UP interventions in addiction community services.

The main aim is to evaluate the pilot implementation process of DBT and UP by the professionals who will decide to implement the interventions after the training.

Specific aims:

1. To evaluate relevant implementation outcomes (acceptability, appropriateness, feasibility, reach, sustainability and barriers/facilitators) after DBT and UP implementation.
2. To assess the professionals’ attitudes toward EBTs, burnout, implementation climate, organizational readiness for implementation and beliefs about the nature of the alcohol addiction after DBT and UP implementation.
3. To evaluate the fidelity of the intervention to be applied by practitioners during the implementation process of DBT and UP.
4. To explore the predictor variables of successful implementation taking into account the moderating or mediating role of the characteristics of the professionals and the organization.
5. To collect barriers and facilitators (based on the CFIR model) that mental health professionals may encounter when implementing DBT and/or UP interventions in order to refine implementation strategy.

### Trial design

This study corresponds to a multicenter pilot implementation study, which allows for assessments of the feasibility, utility, acceptability or quality of research methods for use in a planned definitive trial [65]. This pilot study will evaluate a facilitation implementation strategy at the provider and organizational level for the two transdiagnostic interventions to treat alcohol addiction. A parallel mixed methods design will be used, in which quantitative and qualitative data will be collected simultaneously, analyzed independently and, finally, the results will be integrated together [66].

Regarding quantitative variables, this study presents a reverse counterbalanced (ABBA) intrasubject experimental design [67] where participants will be randomly assigned to first receive DBT or UP online training in phase 1 of the study (and then the other one), and then randomly assigned to implement DBT or UP intervention (or both) in phase two.

As for the qualitative analysis, thematic content analysis will be carried out based on grounded theory [68] to define emerging categories of analysis derived from the focus group data.

### Trial status

Participants` recruitment is in progress for the phase 1 of the study (between June 2024 and February 2025) and “not yet started” for the phase 2 (between May 2025 and June 2025).

### Ethics statement

The study follows the standards of the Declaration of Helsinki and existing Spanish and European Union guidelines for the protection of patients in clinical trials. All participants interested in taking part will sign an online informed consent form. The study procedures were approved by the Ethics Committee associated to the University of Zaragoza (Comité de Ética de la Investigación de la Comunidad Autónoma de Aragón, CEICA, Spain) in April 2024, reference number: PI23–348.

### Participants and recruitment

In phase 1, mental health professionals (psychiatrist, psychologist, nurse and social worker) who work in a community drug addiction service of the Spanish health system in the Aragón, Valencia and Catalonia regions (Spain). The data of the region of each participant will be blindly treated. The following are the inclusion criteria: 1) To be at least 18 years old; 2) To be a mental health professional currently working in a drug dependence care resource; 3) To agree to receive training in DBT and UP; 4) To understand the Spanish language; 5) To accept the informed consent. The exclusion criteria are: 1) Not being interested in receiving training in emotional regulation interventions; 2) Not including in their functions the psychological treatment of people with alcohol addiction; 3) Not having Internet connection to be able to connect to the training sessions.

Recruitment process will be initiated by contacting the health managers of addiction services of each region (Spanish national health system) and asking them for support to recruit the study participants. The data of the region of each participant will be blindly treated. After that, mental health professionals at alcohol addiction centers will receive an email with the research study information and an invitation to participate. Once participants have expressed their interest in participating, the research team sets up an online informative session to explain the research study and answer any questions. The recruitment process in this phase will take place between 1 May 2024 and 31 January 2025. After that, the professionals will receive training and complete the post-training evaluation during February 2025.

Participants in phase 2 will be mental health professionals working in a community drug addiction service in Aragón, Valencia and Catalonia who have received the phase 1 training. The data of the region of each participant will be blindly treated. The following are the inclusion criteria: 1) To be at least 18 years old; 2) To be a mental health professional (psychiatrist, psychologist, nurse and social worker) currently working in a drug addiction community service; 3) To have received training in DBT and UP interventions in phase 1; 4) To agree to implement at least one of the two interventions for alcohol addiction patients and receive supervision; 5) To understand the Spanish language; 6) To accept the informed consent. The exclusion criteria were: 1) Not having an interest in applying and/or receiving DBT and/or UP supervision; 2) Not including in their functions the psychological treatment of people with alcohol addiction; 3) Not having Internet connection to be able to connect to supervision sessions.

Participants will be recruited from among the professionals in phase 1 who have shown an interest in participating in phase 2. The research team will email participants with information about the phase 2 study and will also set up a meeting to answer any questions. This phase 2 recruitment process will take place between 20 February and 20 March 2025. Implementation groups for each therapy will begin in the last week of march. Following this, collection of all study data will be completed on 1 September 2025 and results are expected between 1 October and 31 December 2025.

### Sample size

Taking into account the statistical analyses proposed in this study, the calculation of statistical power using the G*Power program [69] determined a total sample size of at least 130 professionals for phase 1, to achieve at least a statistical power of 90%, with an alpha coefficient of 0.05 and an effect size of 0.25, as recommended by the literature [70,71].

At phase 2, being a pilot implementation study, and taking into account that a randomized clinical trial is expected to be carried out in the future, a sample size of at least 15 professionals per condition (30 participants total) has been estimated (with 90% statistical power and an alpha coefficient of 0.05, following the recommendations for this type of study [72,73].

### Procedures

**Fig 1:**
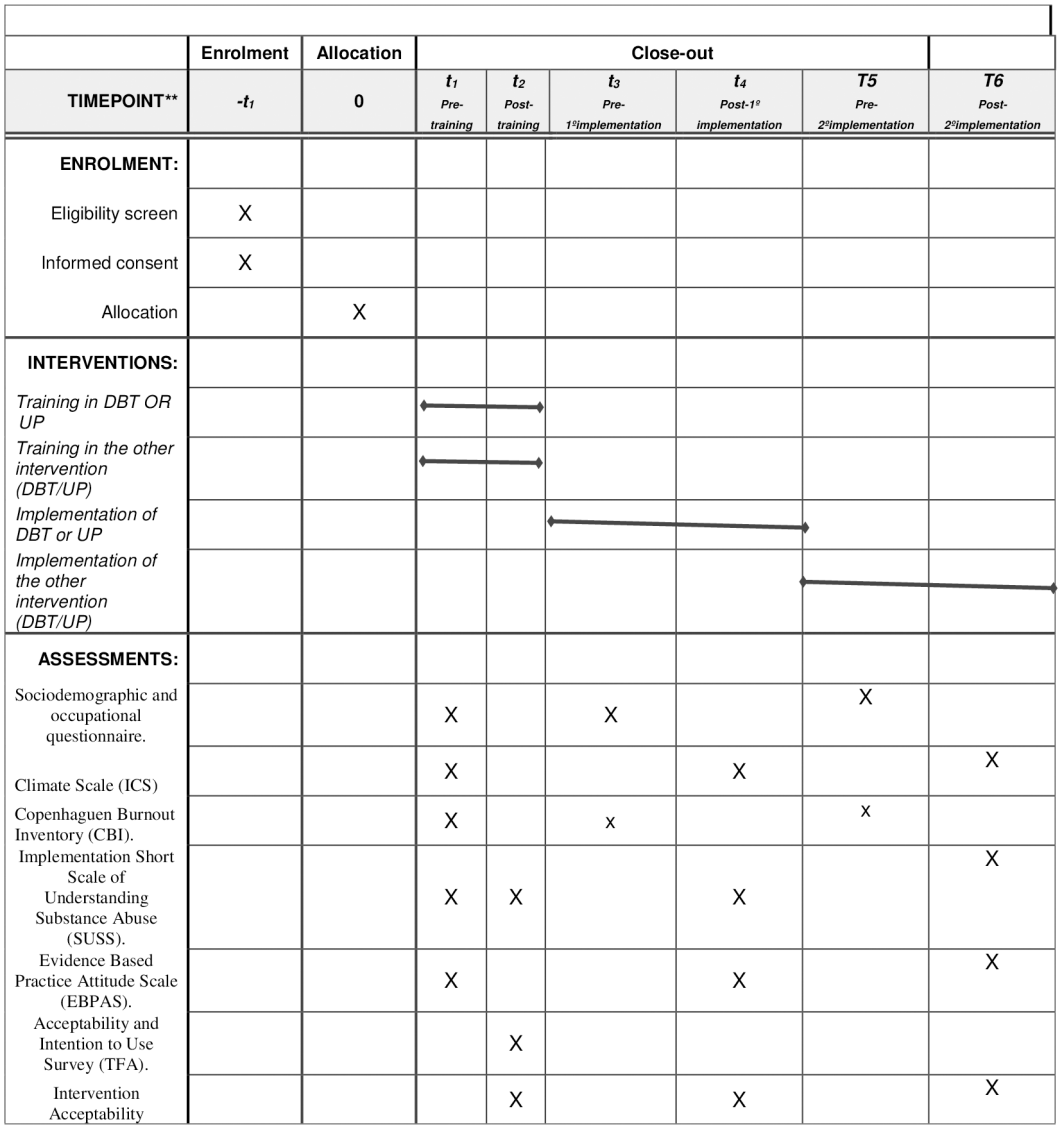
Schedule of enrollment, interventions, and assessments of the study.

**Fig 2:**
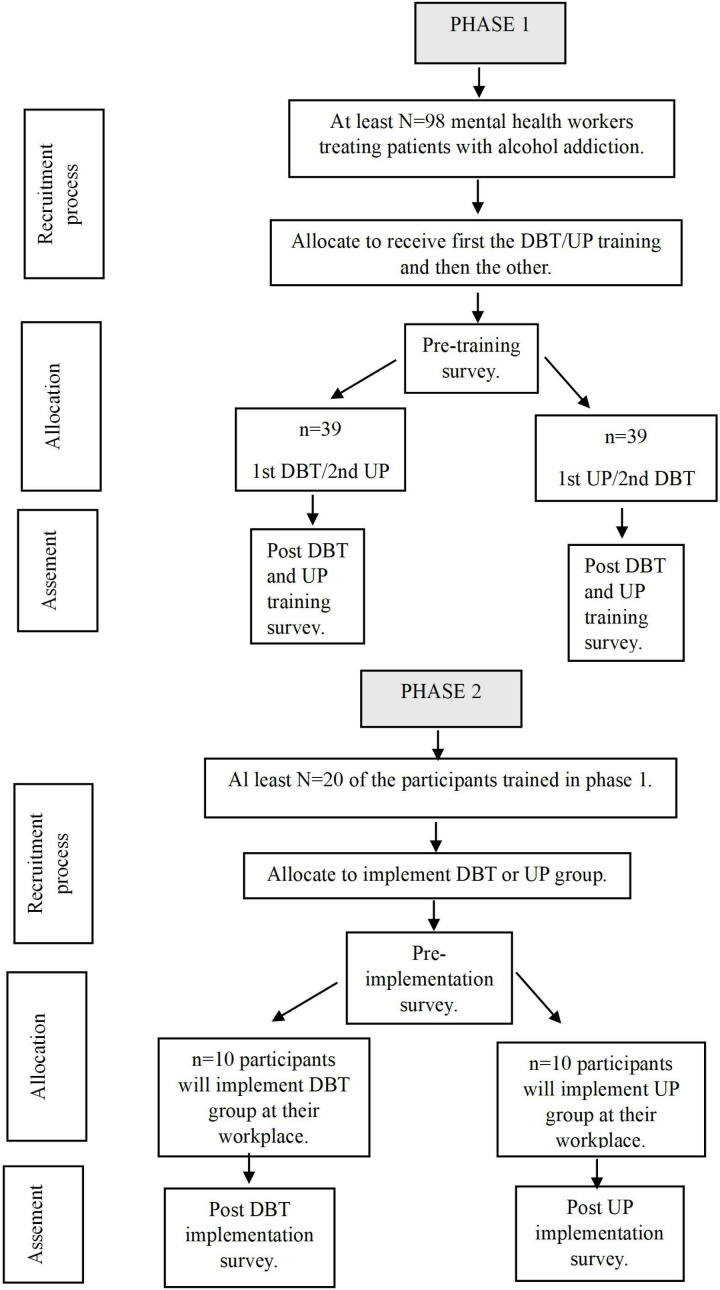
Participants flow chart in phases 1 and 2 of the study. DBT = Dialectical Behavioral Therapy; PU = Unified Protocol.

A schedule of the study enrollment, interventions, and assessments can be found in Fig 1. Fig 2 shows the potential participants flow diagram of the two phases of the study.

Considering the timeline of the study, participant’s recruitment is in progress for the phase 1 of the study (between June 2024 and February 2025) and “not yet started” for the phase 2 (between May 2025 and June 2025). Study data collection, data analysis and obtaining results will take place between February 2025 and May 2025 for phase 1 and between September 2025 and December 2025 for phase 2.

The procedure for each phase of the study is described below:

*Phase 1: DBT and UP training for professionals treating alcohol addiction and pre-implementation.*

Following the recruitment, after reading the study information and signing the online informed consent form via Qualtrics® platform, individuals who agree to participate in the study will be randomly assigned to first receive DBT or UP training (balanced randomization [1:1]), through the randomizer software to guarantee equivalence between the groups. A predoctoral student (blind) that is part of the research team will generate the random allocation sequence and assign participants to interventions. After that, participants will access the online pre-training assessment survey hosted on the Qualtrics® platform (https://www.qualtrics.com/es/) one week before the training.

The training will be carried out in a group and online format and will consist of an 18-hour 3-day workshop for each intervention (DBT and UP) with the following general contents:

*DBT workshop training:* will consist of two blocks. The first block will introduce the key concepts of DBT, justify the suitability of DBT for the treatment of alcohol addiction and present the DBT-SUD program (dialectical abstinence model, attachment strategies, behavioral analysis). In the second block, the content and exercises of the DBT group skills training modules (mindfulness, distress tolerance, addiction and emotion regulation skills) will be presented session by session.

*UP workshop training:* will have also two blocks. In the first one, the justification of the transdiagnostic approach and the adequacy of the UP for the treatment of alcohol addiction will be presented. The dimensional assessment of EDs, the formulation of cases according to the UP, and the main characteristics of the UP will be also explained. In the second block, the content and exercises of the 8 modules of the UP group, including those associated with the 5 ER strategies (mindful emotion awareness, cognitive flexibility, countering emotional behaviors, interoceptive exposure and emotion exposures), will be presented session by session.

At the end of each training course, participants will fill in the post-training survey via Qualtrics. One-two weeks after, they will be invited to participate in a *focus group* discussion in which they will be asked about the advantages and disadvantages of the program and possible adaptations needed to apply each intervention in their actual work settings with patients with alcohol addiction. Focus groups will be conducted until the discourse is saturated [68].

*Phase 2: Pilot implementation of DBT and UP interventions in addiction community services.*

Individuals who agree to participate in the phase 2 of the study will read the study information and sign the online informed consent form. Participants will then be randomly assigned to apply either the DBT or the UP-group intervention first. Those participants who decide to apply both interventions will be able to conduct the second intervention after completing the first one. After filling in the consent form, participants will then access the online pre-implementation assessment survey hosted on the Qualtrics® platform (https://www.qualtrics.com/es/) a week before the implementation.

The implementation of the DBT and UP interventions in phase 2 will be carried out in a group format and will last 3 months (12–14 weekly sessions). A group and brief format have been chosen due to the difficulties implementing long-term treatments in the Spanish health system (e.g., high waiting lists, [[74,75]). The interventions will be adapted according to the context in which they are applied following the qualitative feedback collected in phase 1.

The DBT program is based on an adaptation of the Maffei et al.’s program of DBT group skills training for alcohol addiction [39]. DBT is planned to be implemented over three months, one session a week, lasting 2 hours. For UP, the adaptation of Barlow et al. is will be used [27,43] with the same duration and frequency. Table 1 describes the program contents for each intervention.

**Table 1:** DBT and UP 3-month group intervention sessions to treat alcohol addiction.

Session number	DBT program content	UP program content
1	Orientation and mindfulness skills: Observing, Describing, Participating.	Setting goals and maintaining motivation
2	Orientation and mindfulness skills: Non-judgmentally, one-mindfully, effectively.	Understanding your emotions: What is an emotion?
3	Distress tolerance skills: TIP, distraction, pros and cons.	Understanding your emotions: Following the ARC
4	Distress tolerance skills: Self soothe, IMPROVE skills, radical acceptance.	Mindful emotion awareness I
5	Addiction skills: Dialectical Abstinence.	Mindful emotion awareness II
6	Addiction skills: Clear Mind.	Cognitive flexibility I
7	Addiction skills: Burning bridges and building new ones.	Cognitive flexibility II
8	Addiction skills: Community reinforcement.	Countering emotional behaviors
9	Emotion regulation skills: Identifying and describing emotions.	Understanding and confronting physical sensations
10	Emotion regulation skills: Check the facts, opposite action.	Putting it into a practice: emotion exposures I
11	Emotion regulation skills: Problem solving and mindfulness of emotions.	Putting it into a practice: emotion exposures II
12	Emotion regulation skills: Reducing vulnerability and self-management/rehabilitation.	Moving up from here: Recognizing accomplishments and looking to your future

Group supervisions (2 hours per week) led by the trainer experts will be conducted to monitor the implementation of the therapy sessions. In these supervision sessions, participants will provide comments and constructive criticism on their experience with the previous week’s group and the objectives and tasks for the following session will be prepared. They will also work on the difficulties encountered and possible unforeseen events that may arise during the process of the implementation. This process will be repeated for 12–14 sessions over approximately 3 months via online meetings, ensuring the fidelity of both interventions.

After each implementation process, participants will answer a post-implementation survey via Qualtrics. One-two weeks after, they will be invited to participate in a focus group discussion in which they will be asked about the barriers and facilitators for the implementation process of each intervention. Phase 2 focus groups will also be conducted until the discourse is saturated.

### Outcome measures

Assessment instruments include demographic and professional characteristics (age, sex, education, work status, previous experience by the interventions, etc.) and self-report measures for both primary and secondary outcomes as well as qualitative measures.

Concerning phase 1, the *primary outcome measure* will be the final value of the variable acceptability and intention to use the interventions from the Acceptability and Intention to Use Survey [59,76]. As *secondary outcome measures*, the following will be evaluated: Attitudes towards EBPTS (EBPAS), organizational readiness to implement (ORIC), implementation climate (ICS), burnout (CBI), appropriateness and feasibility (AIM&IAM&FIM), and perceived ability to implement the interventions (EBP-BS) beliefs about nature of alcohol addiction (SUSS), knowledge acquisition of DBT and UP skills and barriers to implementation (BTI).

Regarding phase 2, the *primary measure* will be the total number of practitioners who implement DBT and/or UP group therapy at the end of the implementation phase. As *secondary outcome measures*, attitudes towards DBT and UP, organizational readiness to implement the intervention, implementation climate, burnout, beliefs about nature of alcohol addiction, appropriateness, feasibility and sustainability of the intervention (PSAT), implementation process (NoMAD), barriers to implementation, fidelity of the intervention, and barriers and facilitators to the implementation based on the CFIR model (pCAT) will be evaluated.

Tables 2 and 3 provide a detailed summary of the outcome measures to be used during the two phases of the study. Measures that were used in phase 1 are not described in Table 2.

**Table 2:** Summary of the quantitative and qualitative instruments and assessment time points of phase 1.

Instruments	Authors and adaptations	Variables and Subscales	Psychometric results (Cronbach’s Alpha)	Assessment Time
**Sociodemographic and occupational questionnaire**	Designed ad hoc by the research team.	Age, sex, educational level, marital status, employment status, information on the type of discipline exercised, theoretical orientation and population treated, experience applying DBT and UP, level of DBT and UP training.	Not applicable.	Quantitative. Pre training.
**Acceptability and Intention to Use Survey (TFA)**	Peris-Baquero et al. [59]. Adapted by the team in Spanish.	**Acceptability and Intention to Use** Based on the theoretical framework of acceptability (TFA, Sekhon et al., 2017) model. Nine items composed of seven constructs: Affective attitude; Burden; Ethics; Consistency of intervention; Opportunity costs; Perceived efficacy and Self-efficacy.	Not applicable	Quantitative. Post training.
**Evidence Based Practice Attitude Scale (EBPAS)**	Aarons, Ehrhart, & Farahnak [77]. Spanish version validated by De Paúl, Indias & Arruabarrena [78].	**Attitudes toward Evidence Based Practice (EBP)** Fifteen-item scale composed of the following subscales: Likelihood of adopting EBP given the requirements to do so; Intuitive attractiveness of EBP; Openness to new practices; Perceived divergence of usual practice from EBP.	α>.70 for subscales except “perceived divergence”: α range of.59-.66. This subscale was excluded from this study.	Quantitative. Pre training and post implementation.
**Organizational Readiness for Implementing Change (ORIC)**	Shea et al. [79]. Spanish version developed by ImpleMentAll available in: https://www.implementall.eu/9-outcomes-and-resources.html#ORICtranslations.	**Organizational Readiness for Implementing Change** This 12-item scale consists of the following two subscales: Commitment to change Effectiveness with change	α>.70 for the total scale and its subscales.	Quantitative. Post training and post implementation.
**Intervention Acceptability Measure, Intervention Appropriateness Measure and Feasibility of the intervention (AIM&IAM&FIM)**	Proctor et al. [64]. Spanish back translation was performed.	A**cceptability, appropriateness and feasibility** Consists of three scales measuring each variable with four items each.	Total and subscales were α > .70. Weiner et al. [80].	Quantitative. Post training and post implementation.
**Implementation Climate Scale (ICS)**	Aarons, Ehrhart, & Farahnak. [77]. Spanish back translation was performed.	**Organizational climate** An 18-item unidimensional scale that assesses the degree to which there is a strategic organizational climate that supports the implementation of evidence-based practices.	α>.70 for the total scale and its subscales.	Quantitative. Pre training and post implementation.
**Copenhaguen Burnout Inventory (CBI)**	Kristensen et al. [81]. Spanish version validated by Molinero, Basart & Moncada [82].	**Burnout** Inventory of 18 items. It measures burnout syndrome and it is structured in three subdimensions: personal, work-related and client-related.	α>.70 for the total scale and its subscales.	Quantitative. Pre training and post implementation.
**Short Scale of Understanding Substance Abuse (SUSS)**	Humphreys et al. [83]. Spanish back translation was performed.	**Beliefs about nature of alcohol addiction** Scale of 19 items. It consists of three subscales: disease model, psychosocial model and eclectic orientation.	For the first two subscales α > .70, and low (α = .61) for the eclectic orientation subscale.	Quantitative. Pre and post training and post implementation.
**UP and DBT knowledge acquisition tests**	Ad hoc questionnaires designed by trainers.	**Knowledge acquisition** Ten multiple choice questions with only one correct option.	Not applicable.	Quantitative. Post training.
**Barriers to implementation Inventory (BTI; Behavioral Tech, LLC. (s.f.)**	Landes et al. [51]. Spanish back translation was performed.	**Barriers to implementation** Inventory of 39 items. Structured by the following domains: equipment problems, administrative problems, theoretical/philosophical problems and structural problems.	For the total scale and subscales α > .70. Chugani et al. [84].	Quantitative. Post training and post implementation.
**Credibility/Expentancy questionnaire (CEQ)**	Borkovec & Nau. [85].	**Satisfaction with training** Six-item scale. It evaluates how logical, successful, recommendable, useful, helpful and non-aversive the training is. It also has two other open-ended questions about improvements and strengths of the training.	For the total scale, α > .70. Devilly & Borkovec, [86].	Quantitative. Post training.
**EBP Belief Scale (EBP-BS)**	Melnyk et al. [87]. Spanish back translation was performed.	**Value and ability to implement** A 16-item unidimensional scale that measures the person’s beliefs about the value of EBP and the ability to implement it.	α>.90 for the total scale and its subscales.	Quantitative. Post training.
**Focus group interview**	Designed ad hoc by the research team.	**Advantages, disadvantages, complexity,** **barriers and adaptations** needed for DBT and UP implementation.	Not applicable.	Qualitative. Post training.

**Table 3:** Summary of the quantitative and qualitative instruments and assessment time points of phase 2 (only the new to phase 2 are included).

Instruments	Authors and adaptations	Variables and Subscales	Psychometric results	Assessment time

**Developmental Normalization Questionnaire (NoMAD).**	Finch et al. [88]. Spanish version developed by ImpleMentAll available on: https://www.implementall.eu/9-outcomes-and-resources.html#NoMADtranslations.	**Implementation processes** A 12-item questionnaire. Evaluated from the perspective of the professionals involved. It has four dimensions: coherence of the intervention with daily routine, cognitive participation, collective action of individuals and groups to apply the innovation in daily practice, and reflective follow-up.	α>.70 for the total scale and its subscales.	Quantitative. Pre and post implementation.
****Tools for measuring interventions adherence** **.****	“The TDC Adherence & Fidelity Project” (https://www.TDCadherence.com/) and “Unified Protocol Institute” (http://www.unifiedprotocol.com).	**Fidelity** Several factors are evaluated, such as alignment of session content with the manual, completion of session exercises, incident resolution, therapist skill, patient adherence, and perception of the therapeutic bond.	Not applicable.	Quantitative. During implementation.
**Program sustainability assessment tool (PSAT).**	Luke et al. [89]. Spanish version available on: https://sustaintool.org/psat/assess/.	**Sustainability** A 40-item scale on intervention. The subscales are political support, funding stability, stakeholder partnerships, organizational capacity, program evaluation, program adaptation, communication with stakeholders and strategic planning.	α>.70 for the total scale and its subscales.	Quantitative. Post-implementation
**Pragmatic context assessment tool (pCAT)**	Robinson & Damschroder [90]. Spanish back translation was performed.	**Barriers/facilitators** List of 14 items related to CFIR domains to be evaluated as barriers/facilitators and as relevant or not in the implementation.	Not applicable.	Quantitative. Post-implementation
**Focus group interview**	Designed ad hoc by the research team.	Description of **barriers and facilitators** found in the different domains and solutions to overcome them.	Not applicable	Qualitative. Post-implementation

### Data analysis plan

Intention-to-treat analyses will be conducted with all participants (completers and non-completers). In phase 1, quantitative data will be analyzed using SPSS V25.0 statistical software [91]. First, descriptive statistical analyses will be conducted to analyze the characteristics of the participants. Subsequently, analysis of variance (MANOVA) or Chi-square tests will be carried out to explore the existence of differences (sociodemographic and in the variables under study) between participants who receive DBT training first and those that get UP training and between the results of both interventions, and also between completers and non-completers. Similarly, linear mixed models will be used to explore the changes that occur during the different evaluation moments. These models are efficient when there is a possibility of working with missing data, which frequently occurs when working with successive measurements from the same participants over time [92]. Finally, the analyses will include the calculation of effect sizes (*Cohen’s d*), to assess the magnitude of change. A subgroup analysis by gender will be included in the analysis and interpretation of results. For qualitative data analysis from focus groups, the MAXQDA [93] statistical program will be used. Following Schreier’s procedure, transcribed data will be analyzed, with categories, subcategories, and areas extracted by two independent researchers to ensure reliability [68]. Disagreements will be resolved through researcher triangulation, involving a third researcher if needed. Cohen’s Kappa will be calculated to assess agreement between the initial and final versions of information extraction.

In phase 2, descriptive statistical analyses will be carried out to analyze the characteristics of the participants. Then, depending on whether the sample follows a normal distribution, Chi-square, T-student and analysis of variance (MANOVA) tests will be performed to explore statistically significant differences in the study variables among participants (including comparisons between completers and non-completers) and, over time, of comparable nonparametric analyses. Pearson’s R correlations will also be carried out to analyze relationships between the study variables. Finally, multiple linear regression analyses and mediation and moderation analyses will also be conducted to identify predictor, mediator and moderator variables of the results. All analyses will be carried out using SPSS V25.0 statistical software [91] and Mplus V.8.0 [94].

## Discussion

The general goal of this study is to evaluate the effect of a dissemination and pilot implementation initiative of DBT and UP programs on healthcare workers treating alcohol addiction in the Spanish health system. The study has been divided in two phases.

In phase 1, the effect of DBT and UP trainings in mental health professionals treating people with alcohol addiction will be evaluated.

DBT intensive training has been considered one of the most successful dissemination efforts by those developing EBPTs [95]. Current literature suggests good effectiveness results of this training to implement DBT among trained professionals [e.g., 48,75]. DBT intensive training also helps improve knowledge, motivation, competence and perceived self-efficacy to apply DBT among professionals [e.g., 36] and decrease therapist´s stress and burnout [e.g., 51,76,96]. Regarding UP, the evidence shows high intention to use this intervention among professionals who had received the training [58]. In a study, positive attitudes towards EBPTs and fewer years of clinical practice were associated with higher satisfaction with UP. Findings also showed that, after participating in the training workshop, practitioners showed good understanding of UP treatment concepts [56].

To the best of our knowledge, no studies have been focused on evaluating the implementation variables proposed in this study in Spanish-speaking professionals treating alcohol addiction. Taking into account the findings of previous studies, we expect to find high acceptability, feasibility, appropriateness and intention to us both interventions by professionals, as well as to observe significant effects on other therapist characteristics.

Additionally, one of the specific objectives set for phase 1 is to collect the opinions from mental health professionals for the adaptations needed so that the DBT and UP can be implemented at their workplace. This qualitative information may allow to expand the knowledge to successfully adapt the interventions before their implementation is carried out in routine clinical practice of the addiction community services.

In the second phase, after professionals have received the specialized training, the pilot implementation process of DBT and UP group programs by the professionals in routine clinical practice will be evaluated.

Scientific literature has explored possible barriers and facilitators encountered by professionals when implementing DBT. Main barriers include the lack of organizational and peer support and the staff turnover. The most relevant facilitators are: greater prior experience and training on DBT, effective leadership and the possibility of being supervised [50,51,97–99]. Literature on the implementation of the UP is scarce, but there is some incipient data that suggest that barriers traditionally encountered in the implementation of other EBPTs could be extrapolated to this intervention [56].

This current study expects to find implementation results (e.g., significant scores of acceptability, appropriateness, feasibility, reach, sustainability and fidelity) similar to those found in previous studies with English-speaking professionals. It may be that new and different barriers and facilitators arise, since no previous studies have evaluated the implementation of DBT and UP in community addiction services. The barriers and/or facilitators detected will serve to identify the type of resources/professionals and/or teams where such implementation efforts are most likely to succeed. All of this information may contribute to reducing the great time lag those effective interventions take to be implemented.

To our knowledge, this will be the first study evaluating the effects of dissemination and pilot implementation of DBT and UP to treat alcohol addiction in Spanish community drug addiction services. However, some limitations should be considered in the interpretation of future findings of this study.

Firstly, the study will be carried out mainly in public centers within the Spanish health system, so the results cannot be generalized to other health contexts. Despite this limitation, the results of the study could lay the foundations for factors to be considered to successfully implement DBT and UP for alcohol addiction, so that health managers and mental health professionals working with this population in other contexts can replicate the study. Secondly, given that participation is voluntary and requires considerable availability and effort, difficulties in recruiting the total sample size needed for the two study phases may be encountered. If this were to happen, the inclusion of participants from other regions of Spain could be considered. In addition, informative meetings will be held with interested participants to clarify expectations and create a collaborative environment that will encourage the professionals’ participation. Thirdly, although innovative validated measures have been included in this study to evaluate relevant suggested implementation variables, some of the questionnaires that will be used are not yet validated for its use in Spanish. This could reduce the validity of some of the conclusions obtained. To avoid this problem, back-translations have been carried out and it is planned to validate these measures in future studies. Finally, client-level outcomes are not included in this initial study. Client outcomes during the implementation process are relevant to obtain concurrent data that continue to demonstrate the efficacy of interventions once implemented in real and adaptive settings and to link more traditional clinical research (efficacy measures) with end-of-life care services and their needs. Therefore, although it has not been possible to access this information in this pilot study, hybrid implementation trials will be [100] conducted in subsequent studies.

Despite the limitations, this study presents a number of benefits for future research. Firstly, if interventions can be implemented successfully, this would have an important impact on the Spanish Health System by reducing the time and resources invested in training mental health professionals. By offering group format interventions, more people with alcohol addiction may benefit, thus reducing waiting lists. Secondly, the transdiagnostic treatments offered have been scientifically contrasted and are usually accompanied by treatment manuals, which would facilitate treatment work and therapists’ fidelity to the principles and techniques of the interventions. Finally, this research presents a high degree of translation. The context where the study will be carried out is in the addiction services from different regions of the Spanish health system, which implies that findings from this study will ultimately benefit different agents of the community such as managers, directors, mental health professionals, patients, their families and as a consequence, society.

## Supporting information

S1 Fig SPIRIT checklist. (DOCX)

S2 Protocol Study Protocol approved. (PDF)
