# Supplementary material for: Implementation of two transdiagnostic interventions based on emotional regulation for professionals who treat alcohol addiction in the Spanish mental health system: A multicenter mixed methods pilot study protocol
Source: PLoS One. 2025 May 9;20(5):e0318512. doi: 10.1371/journal.pone.0318512 (PMC12064197; doi:10.1371/journal.pone.0318512)
Supplement: S2 Protocol — (PDF) [file pone.0318512.s002.pdf]

CEICA RESEARCH PROJECT PROTOCOL

**TITLE**	**[2023I060] Dissemination, acceptability and implementation study of two transdiagnostic psychological interventions based on emotional regulation for the treatment of alcohol addiction: Dialectical Behavioral Therapy and Unified Protocol.**
**VERSION AND DATE**	V1.26.02.2024
**CENTER WHERE THE STUDY IS PERFORMED**	Faculty of Social and Human Sciences (University of Zaragoza, Teruel Campus) Funded by the National Plan on Drugs, Ministry of Health.

**PRINCIPAL INVESTIGATOR OF THE PROJECT**	
**NAME AND SURNAME**	María Vicenta Navarro Haro
**ID CARD**	
**E-MAIL* E-MAIL* E-MAIL* E-MAIL* E-MAIL* E-MAIL**	
**TELEPHONE*.**	
**WORKSTATION**	Associate Professor
**SERVICE/DEPARTMENT**	Department of Psychology and Sociology
**CENTER/FACULTY/UNIVERSITY / OTHER**	Faculty of Social and Human Sciences. Teruel Campus. University of Zaragoza

**PROMOTER (Essential for clinical trials and observational drug studies)**	
**COMPANY IDENTIFICATION/ FIRST NAME AND SURNAME*.**	Plan Nacional Sobre Drogas (Ministerio de Sanidad, Spain)
**VAT ID*.**	
**E-MAIL* E-MAIL* E-MAIL* E-MAIL* E-MAIL* E-MAIL**	sdgpnsd@sanidad.gob.es
**TELEPHONE*.**	

**CONTACT OF THE PERSON IN CHARGE OF HANDLING THE REQUEST**<br>*If you wish, please add the contact details of the person in charge of handling the details of the request.*	
**NAME AND SURNAME**	María Vicenta Navarro Haro
**E-MAIL**	mvnavarro@unizar.es
**TELEPHONE**	

The personal data that may be contained in this communication will be incorporated into the processing system for which the Aragonese Institute of Health Sciences (IACS) is responsible. The data will be processed for the management and follow-up of the

studies evaluated by CEICA. The data will be deleted when the management and/or processing of the request has been answered and is no longer necessary. You have the right to access, rectify and delete the data, as well as other rights.

The data protection regulations of the IACS, domiciled at the Centro de Investigación Biomédica de Aragón. Avda. San Juan Bosco, nº 13, 500009, Zaragoza or by sending an e-mail to protecciondedatos.iacs@aragon.es.

GLOSSARY

- Anonymization: process by which it is no longer possible to establish by reasonable means the linkbetween a piece of data and the subject to which it refers. It is also applicable to the biological sample.
- BIGAN: health big data platform (managed by IACS) that allows access to Aragonese Health System data in a pseudonymized way for its use in management and research.
- Biobank: public or private, not-for-profit establishment that houses one or more collections of biological samples of human origin for biomedical research purposes, organized as a technical unit with criteria of quality, order and destination.
- Center: Institution where a study is conducted (hospital, health center, residence, faculty, private clinic, school, etc.). **In case of doubt, reference should be made to the place where the participants come from** (hospital, school, sports club, etc.).
- Confidentiality Commitment: Document that, on a mandatory basis, must be signed by students and residents who carry out any activity in the public health system (model established in Order SSI/81/2017).
- Informed consent: manifestation of free and conscious will validly issued by a capable person, or by his/her authorized representative, preceded by adequate information.
- CEICA opinion: document that certifies that the CEICA has evaluated a research project and that the project complies with the applicable legal norms and ethical criteria.
- Survey: collection of information in physical or digital support, with or without direct interaction with the source subject.
- Interview: collection of information with direct interaction with the source subject, through verbal responses.
- Research team: A group of researchers who jointly carry out a specific project.
- Primary data source: When data are collected directly from the study participant and for the purpose of the study.
- Secondary data source: When data already collected (and therefore recorded) that were obtained for a purpose other than the study (welfare, teaching, etc.) are used for the study.
- Research group: A group of researchers with a common trajectory (publications, financing) directed or coordinated by a Principal Investigator, grouped around a research topic and not necessarily linked to a health care or departmental affiliation.
- Participant information sheet: Document informing potential participants of the nature of the study, so that they can give their informed consent.
- Intervention (intervention study): any action to be performed on a person due to his/her participation in a study (it can be a pharmacological, physiotherapeutic treatment, an educational, behavioral, psychological intervention).
- Principal Investigator: Investigator who leads the project and is responsible for its design, conduct and dissemination of the results. If the study is multicenter, there should be a principal investigator in each center who is responsible for the patients, data and/or samples.
- Biological sample: any biological material of human origin that can be preserved and that may contain information on the characteristic genetic endowment of a person.
- Routine clinical practice: Procedures that are carried out for purely health care purposes, regardless of whether or not an individual participates in a research study.
- Research project: scientific procedure aimed at gathering information and formulating hypotheses on a given social or scientific phenomenon.
- Promoter: individual, company, institution or organization responsible for initiating, managing, organizing and financing a study.
- Pseudonymization: the processing of personal data in such a way that they can no longer be attributed to a data subject without the use of additional information, provided that such additional information is separately identified and subject to technical and organizational measures designed to ensure that the personal data are not attributed to an identified or identifiable natural person.

SCOPE AND FINANCING OF THE PROJECT	
Is it a multicenter project? Yes ☒ No ☐	If yes, the complete list of centers must be submitted and a team commitment must be filled out. researcher **for each center in Aragón** (see Annex I).
Is specific funding available for the study? Yes	In **all cases,** Annex II: Authorization for use of resources must be completed. In addition, if yes, the budget and source must be submitted. of financing.

2. CHARACTERISTICS OF THE STUDY	
**2.1 Is this research involving drugs** ☐	Yes ☐ No ☒ Yes ☒ No ☒ No
If yes, please select one option:<br><br>1) ☐ This is an observational study regarding drug treatment (EOM) In this case, specify:<br>☐ Prospective data collection ☐ Retrospective data collection ☐ Cross-sectional data collection.<br><br>2) ☐ This is an intervention study: clinical trial with drugs.<br>In this case, it must be submitted according to AEMPS instructions (https://www.aemps.gob.es/medicamentos-de-uso-). human/investigacionclinica_medicamentos/ensayosclinicos/#n-en-espanola)	
**2.2 Is this an investigation involving medical devices or medical devices**?	Yes ☐ No ☒
If yes, please select one option:<br><br>1) ☐ This is an observational study regarding the use of the medical device.<br><br>2) ☐ It is an intervention study: clinical trial with medical devices In this case it must be submitted according to CEICA's SOP for this type of studies (https://www.iacs.es/investigacion/comite-de-etica-de-la-investigacion-de-aragon-ceica/).	
**2.3 Is this research involving invasive procedures?**<br>(Definition: any intervention performed for research purposes that involves physical or psychological risk to the participant).<br><br>If so, an insurance policy must be taken out or a minimum risk must be justified.	Yes ☐ No ☒ Yes ☐ No<br>☒ Yes ☒ No ☒ No
**2.4 Does the research involve minors or persons incapable of giving consent?**<br><br>If yes, an information and informed consent document addressed to the guardian/legal representative/family member and another addressed to the minor must be submitted. (adapted to their capacity). Review CEICA template.	Yes ☐ No ☒ Yes ☐ No<br>☒ Yes ☒ No ☒ No

2. CHARACTERISTICS OF THE STUDY	
**2.5 Are biological samples used in the study?**	Yes ☐ No ☒ Yes ☐ No ☒ Yes ☒ No ☒ No
If yes, please select one or more options:<br><br>1) ☐ Samples of surplus care are used with consent for the project The information and consent document (CEICA template) must be submitted.<br><br>2) ☐ Surplus care samples are used without consent.<br>It must be adequately justified in the section on ethical aspects (art. 58.2 Law 14/2007).<br><br>3) ☐ Samples are collected specifically for this study.<br>The information and consent document (CEICA template) must be submitted.<br><br>4) ☐ A private sample collection is created.<br>For the creation of a new collection, submit the necessary documentation for evaluation and indicate the registration number (no.) (see CEICA website).) (see CEICA website)<br><br>5) ☐ Samples already collected in a private sample collection are used.<br>In this case, identify the collection number and the person in charge:___________<br><br>6) ☐ Samples are requested from an authorized Biobank. In this case, identify the Biobank:______<br>The request must be submitted to the biobank	
**2.6 Is genetic analysis performed?**	Yes ☐ No ☒ Yes ☐ No ☒ Yes ☒ No ☒ No
**2.7 Are embryos, human embryonic cells, human fetal cells or tissues or human pluripotent cells obtained by cell reprogramming used?**<br>You should contact IACS or the responsible institution for further approvals.	Yes ☐ No ☒ Yes ☐ No ☒ Yes ☒ No ☒ No

3. PROCESSING OF PERSONAL DATA

Personal data is considered to be any data (age, sex) or any information (numerical, alphabetical, graphic, acoustic) on an identified or identifiable natural person; any person whose identity can be determined, directly or indirectly (i.e. who has not been irreversibly anonymized at source) is considered to be identifiable.

**3.1 Is personal data collected or processed in the study?**	Yes ☒ No ☐ Yes ☒ No ☐ Yes ☒ No ☐ No ☑ No

If yes, check the applicable option:

☒Informed consent is requested from the data subject.
Submit the information and consent document (CEICA template).

☐Data obtained for another purpose that have been pseudonymized (e.g., medical history, other research, other records) are used in accordance with A.D. 17 of Organic Law 3/2018.

☐Other. Specify:

Check the **categories** to which the collected data belong:

Identifying data (name, address, email, DNI, medical record number, telephone, signature, IP, geolocation, image/voice, other)

☒ **personal data:** date of birth, place of birth, parents' names, place of work, economic data, sex, marital status, children, academic qualifications, other.

☒ opinion data

☐ **particularly sensitive data**: health, ethnicity, religion, political opinion, sexual life or orientation, trade union membership, special educational needs, etc.

3.2 If the data are collected directly from the data subject (primary source), specify the procedure (Example: interview, paper survey, email, phone, web applications, ...)

The data is collected through online surveys created on the Qualtrics platform (web application). The online survey platform Qualtrics ® complies with the RGPD (General Data Protection Regulation), has an ISO 27001 security certificate and allows the correction, modification and deletion of personal data permanently from its servers. Here is the privacy policy: https://www.qualtrics.com/uk/platform/gdpr/

3.3 If not collected directly from the data subject (secondary source), check the option and specify:

☐ Data from other similar research, for which the consent of the interested party was obtained, are reused. Permission from the person responsible for the data, the consent form with which they were obtained and the commitment to use the pseudonymized data must be submitted (see on the web).

☐ Data obtained for another purpose and without consent are used for research (e.g., medical history or other record).

In this case, indicate:

☐ The investigator (if the investigator is staff of the center) has direct access to the clinical history. Present permission from the person responsible for the data (if it is the clinical history, authorization from the management must be presented for access to data for this study).

☐ The researcher receives the data already pseudonymized Present the commitment to use the pseudonymized data (see on the web).

Note: **researchers who do not have an employment relationship with the hospital/center** do not have access to the medical records, so ALWAYS use this source to obtain pseudonymized data.

In all cases, explain: origin of the data, person in charge of them

☐ Data from BIGAN
Submit Biocomputing Unit report (link to application) and commitment to use the data.
pseudonymized (see on the web)

3.4 Once the information and data have been obtained, how is the privacy of the participants guaranteed?

☐ Only aggregated data are used (i.e., data that correspond to groups of people and not to each individual).

☐ The data is anonymized (the data cannot be associated with an identified or identifiable person because the link to any information that identifies the subject has been irreversibly destroyed).

☒ data are pseudonymized or coded (direct identifiers are replaced by a code/pseudonym known only to the research team).

Explain how and by whom the adopted measure is carried out:
The processing of personal data will be carried out using techniques to maintain anonymity through the use of random alpha-numeric codes, so that personal identity is completely hidden during the research process.

3.5 Data conservation period: specify date of destruction: Until the publication of the results (expected by the end of 2026, since the project would be carried out from January 2024 to December 2025).
It is generally considered appropriate to retain until publication, otherwise, justification must be provided.

3.6 Data Processors (do not fill in in case of anonymous data)

A **processor** is any natural or legal person, public authority, service or other body that processes personal data on behalf of the controller.
If a third party (outside the institution) processes project data, it will be necessary to sign a **processor contract**. A model can be downloaded from https://seguridad.salud.aragon.es/plantillas/

Indicate which persons will process the data collected, specifying who will have access to the identifying data:

Basic information on data protection:
Data controller: University of Zaragoza Internal data controller:
María Vicenta Navarro Haro
Purpose: Your personal data will be processed exclusively for the research work referred to in this document.
Legitimation: The processing of the data in this study is legitimized by your consent to participate. Recipients: No data will be transferred to third parties unless required by law.
Rights: You may exercise your rights of access, rectification, deletion and portability of your data, limitation and opposition to its processing, in accordance with the provisions of the General Data Protection Regulation (RGPD 2016/679) to the principal investigator of the project, and may obtain information about it by sending an email to the address mvnavarro@unizar.es, or by sending an email to the Data Protection Delegate of the University of Zaragoza (dpd@unizar.es). If your request is not answered, you may complain to the Spanish Data Protection Agency (https://www.aepd.es).
Additional information on data protection at the University of Zaragoza is available at the address:
https://protecciondatos.unizar.es/.

The processing of your personal data will be carried out using techniques to maintain your anonymity through the use of random codes, so that your personal identity is completely hidden.
during the research process. Based on the results of the research work, scientific communications may be prepared to be presented at congresses or scientific journals, but they will always be done with grouped data and nothing that could identify you will ever be disclosed.
The Unizar data processing approval document is attached.

Are all persons authorized to process the data subject to a confidentiality agreement signed with the center?

Yes ☒ No ☐ Yes ☒ No ☐ Yes ☒ No ☐ No ☑ No

3.7 Will data be transferred to third parties? Yes ☐ No ☒

In case of assignment, it must be specified:

- the data given up are: ☐ identified, ☐ pseudonymized, ☐ anonymized.

- to whom they are assigned:

- what data is transferred:

- for what purpose:

- Explain how data are pseudonymized or anonymized:

- If there are international transfers: specify company and country (in this case, the express consent of the participant must be requested for this transfer).

PROCESSING OF PERSONAL DATA	
**3.8 Are recordings (audio/video) to be made?**<br>Remember that the express consent of the interested party is required to perform the following activities<br>recordings, this information must be included in the consent document.	Yes ☒ No ☐ Yes ☒ No ☐ Yes ☒ No ☐ No ☑ No

In case of recording, it must be specified:
Recordings of the two focus groups will be made.

- Where they are to be kept, who has access and the security measures to be applied:

In order to safeguard the identity of the participants in the focus groups, the analysis of the information (e.g., extraction of the main themes discussed during the session) will be carried out anonymously. The particular opinion of each participant will not be identified, but global analysis of the information will be carried out. Neither the transcription nor the publication of results will include data that would allow the identification of the professionals. Each participant will be identified with an alphanumeric code (PROF1, PROF2, PROF3, etc.). In addition, after the transcription of the information, the audiovisual recording will be deleted.

- Time period for preservation of the recordings:

After transcription of the information and at the latest at the end of the project.

- for what purpose:

Collect qualitative information in focus groups.

- if computer applications or cloud storage are used, who the service provider is and where it is legally resident, as well as the link to its privacy policy, should be indicated:

The Zoom Platform will be used for the focus group recordings. The privacy policy is described here: https://explore.zoom.us/es/gdpr/

3.9 Information security measures: description of the computer systems to be used

It is reminded that servers containing personal data must be located in the territory of the EU (RDL 14/2019).

- System in which the data is to be stored (personal computer, corporate servers, external company or organization, cloud service provider, etc.)

Physical servers of the University of Zaragoza

- Applications to be used for data processing (excel, spss, etc.)

For the analysis and introduction of quantitative data, the statistical package IBM SPSS Statistics version 22.0 for Windows (IBM Corp., 2013) will be used. For the qualitative analysis of the information obtained through the focus groups, the MAXQDA statistical program also located in the University of Zaragoza devices will be used. First, the word-by-word transcription of the focus groups will be carried out. Based on the procedure described by Schreirer (2012), the transcribed information will be analyzed. This procedure allows the construction of a hierarchical coding system that allows the extraction of "general categories", which are composed of "subcategories" and their corresponding "areas" or "themes". In order to ensure reliability in the information extraction process, the extraction of the "categories", "subcategories" and "areas" will be carried out by two independent researchers of the team. In case of disagreement, a third researcher will be consulted (researcher triangulation).

The data collected for the study will be identified by an alphanumeric code and only the research personnel in the study will have access to them and they will be stored in the devices of the University of Zaragoza.

- If online computer applications or "cloud" storage is used, it should indicate who the service provider is and where their legal residence is located, as well as the link to their privacy policy.

The online survey platform Qualtrics ® complies with the RGPD (General Data Protection Regulation), has an ISO 27001 security certificate and allows the correction, modification and deletion of personal data permanently from its servers. Here is the privacy policy: https://www.qualtrics.com/uk/platform/gdpr/. The Zoom platform will also be used for the

videoconferences of the focus groups. It also complies with regulations. Here is the privacy and data protection policy: https://explore.zoom.us/es/gdpr/

3.10 Information Security Measures: Devices

- Indicate whether any type of removable device (portable USB, external hard disk, etc.) is to be used and whether it is to be encrypted.
- If corporate computer systems are not used, indicate whether backups are made.
- Indicate the security measures for paper documents (custody, access).

No removable devices or paper-based information will be used.

General recommendations on data use

- Do not use Wifi networks to transmit sensitive information.
- Use strong passwords and change them periodically.
- Always encrypt sensitive information to be sent by e-mail.
- Ensure that the versions of operating systems and applications are always up to date.
- On personal computers, always use antivirus software and keep it up to date.
- Never open files attached to e-mails in which you do not identify the sender.
- Do not use social networks to communicate sensitive information.
- The cell phone is not a very secure device for handling sensitive information, and the antivirus programs that can be installed offer little protection.
- Use corporate applications whenever possible
- The use of USB or other removable devices is strongly discouraged.

4. DESCRIPTION OF RESEARCH PROJECT (complete the fields or attach complete protocol with equivalent information)

4.1 Tasks of the research team

Briefly explain who is participating in the study, in what capacity and what tasks they will perform, as well as their affiliation (current job title). The cv and signature of all of them must be submitted in Annex I.
If the study is multicenter, an Annex I must be submitted for each center.

This is a project funded by the Plan Nacional Sobre Drogas, Ministerio de Sanidad (study grant document attached).
The team will consist of four members of the research team and three members of the work team. Their roles are described below.

Research team:

-MVNH (Principal Investigator) is a trainer, supervisor and official therapist in Dialectical Behavior Therapy (by the Linehan Board of Certification). She works as a professor at the University of Zaragoza and is an effective member of the consolidated research group of the Government of Aragon "S31_20D Investigación En Comportamiento, Salud Y Tecnologías (ICST)" and of the research group of the Instituto de Investigación Sanitaria de Aragón "Investigación en Personalidad, Emoción y Salud; IPES). She will be the principal investigator of the project and will be involved in all the tasks of the project, although more specifically she will be in charge of the training and supervision in DBT and in the coordination of the research and work team and the follow-up of the tasks and schedule established in the project.
-JO (Member of the research team) is full professor at the University of Zaragoza, effective member of the consolidated research group of the Government of Aragón "S31_20D Investigación En Comportamiento, Salud Y Tecnologías (ICST)" and director of the research group of the Instituto de Investigación Sanitaria de Aragón "Investigación en Personalidad, Emoción y Salud; IPES). He is supervisor, therapist and trainer in Spanish language of the Unified Protocol Institute (UPI; Boston University, USA). Will be a trainer and supervisor in Unified Protocol (UP) during the study and will participate in the development of the study protocol, sample collection, interpretation of results, and dissemination of the same.

-ECD (Member of the research team) has a PhD in psychology, is an associate professor at the University Jaime I and works in the Section of People with Functional Diversity and Mental Health at the Conselleria de Igualdad y Políticas Inclusivas (Regional Ministry of Equality and Inclusive Policies). She is also a member of the research group of the Health Research Institute of Aragon "Research in Personality,Emotion and Health; IPES). She has been certified as a trainer and researcher in PU by the UPI and has been certified in DBT by DBT Iberoamerica. She will also be a trainer and supervisor in PU and DBT during thestudy and will participate in the collection of the sample, in conducting interviews and collecting the results in the focus groups, and in the interpretation of the results and their dissemination.

-VMB (Member of the research team) has a PhD in Design, Management and Evaluation of Social Welfare Public Policies. She works as a senior research support technician at the Institute for Health Research of Aragon and is an associate professor at the University of Zaragoza. On September 1, 2023 she will sign the position she has obtained at the University of Zaragoza as Assistant Professor. She is also a member of the IPES research group. She has done training (level I) in PU and has applied in PU in people with medical illnesses and comorbidity with emotional disorders, both for prevention and treatment (women with infertility, people with persistent COVID-19). He/she will participate in conducting interviews and collecting results in focus groups, interpretation of results and dissemination of results.

Work team:

- ÓPB (Research staff in training hired without charge to the subsidized project). He is trained and accredited as a PU therapist (Level II, UPI), his doctoral thesis deals with the cost-effectiveness and acceptability of PU in group format in the NHS. Currently, he works as a research staff in predoctoral training at the University of Zaragoza and is an effective member of the group of the Government of Aragon "S31_20D Investigación En Comportamiento, Salud Y Tecnologías (ICST)" and of the group of the Instituto de Investigación Sanitaria de Aragón IPES. Due to his familiarity with the subject and research experience he will participate in the construction of the database and management of the survey platform, as well as in the sample collection and analysis of the results.
- AB; Collaborating Scientific Staff) is a doctor in psychology, an expert in addictions and works as a psychologist at the Amigó Foundation (Proyecto Hombre) in Castellón and as an associate professor in the Department of Medicine and the Department of Social and Legal Sciences at the CEU Cardenal Herrera University.
- LM; Collaborating Scientific Staff) has a PhD in psychology, is an expert in addictions and works as a clinical psychologist at the Center for care and monitoring of drug addictions in Girona and is an associate professor at the University of Girona.

The collaborating scientific staff (AB and LM) will assist with their extensive clinical and research experience in addictions in the development of the research protocol, in the detection of barriers and facilitators for the implementation of the interventions, as well as in the interpretation of the results and in the scientific dissemination of the results.

Due to the fact that this project has been funded with a grant for additions from the National Plan on Drugs, a pre-doctoral trainee was also hired; Alba Abanades Morillo (AA). The specific tasks of each member are described in the work plan under point 8.

4.2 Justification of the study: Background, current state of the subject, relevance (Cite bibliographic references in the following section).

Background and current status of the subject:

Alcohol Use Disorder

Alcohol use disorders (APA (1), including harmful drinking, are fairly common (and largely untreated) mental health disorders associated with significant morbidity and mortality (2,3). According to WHO (4), harmful alcohol consumption is a causal factor in more than 200 diseases and disorders. Each year, 3 million deaths occur worldwide due to harmful alcohol consumption, accounting for 5.3% of all deaths. In addition, harmful use of alcohol leads to significant social and economic losses to individuals and society in general. This occurs at older ages

Among people aged 20 to 39 years, approximately 13.5% of all deaths are attributable to alcohol. There is a causal relationship between harmful alcohol consumption and a number of mental and behavioral disorders. Alcohol dependence is among the most frequent mental health problems in Spain, affecting more than 4% of the population (5), especially in outpatient drug dependence units of the SNS (e.g. about 44% of patients in Catalonia) (6). Patients with alcohol addiction often suffer from other comorbid disorders that complicate treatment, such as personality disorders and emotional disorders (ETs hereafter; anxiety, depression and related disorders) (7). Improving the success rates of intervention programs has generated great research interest (8).

Emotional dysregulation and alcohol addiction

The scientific literature demonstrates that using alcohol as a coping mechanism for distress is a maladaptive emotion regulation strategy. For example, some studies have revealed that mood and negative valence emotions are one of the most important determinants of craving and relapse in people with alcohol addiction (9,10). In fact, in one paper emotional regulation (ER) difficulties were found to predict severity of alcohol use disorder (11). Research suggests that people with alcohol addiction have greater use of maladaptive ER strategies compared to controls (12). In the same sense, it has also been found that they also present higher scores in neuroticism (13) and negative emotionality (14) compared to individuals without alcohol addiction. These findings have raised the possibility that people with addiction might use alcohol as a coping mechanism in response to intense unpleasant emotions (15). For these reasons, working on ER could represent a promising target for the improvement of psychological treatments for alcohol addiction.

Transdiagnostic interventions to treat alcohol addiction

The literature points out that comorbidities associated with the diagnosis of addiction negatively influence treatment outcomes (8). In the last decade, new transdiagnostic treatment proposals have emerged. These interventions focus on SR as a key component of treatment, allowing different comorbidities to be addressed at the same time (16). Evidence supports the notion of emotional dysregulation as a transdiagnostic construct (17-20) across TEs (depressive disorders, anxiety disorders, eating disorders, substance use disorders and borderline personality disorder) (17,21-24). Existing transdiagnostic therapies provide a comprehensive working model in which substance use is seen as a means of addressing affective dysregulation
(16). Dialectical Behavior Therapy (DBT) (25,26) and the Unified Protocol (PU, 27) are two examples of transdiagnostic treatments to address emotional dysregulation in different psychological disorders with good results (28-31).
Below, we discuss the main results of the two interventions to treat emotional dysregulation in alcohol addiction.

1. Dialectical Behavioral Therapy:

DBT was specifically designed for multidiagnosed, high-risk individuals characterized by persistent emotional dysregulation (32). The intervention consists of a combination of individual psychotherapy, group skills training, telephone coaching, and a consultation team with the therapist (25,26). In Spain, the PI of this project has participated in the line of research proposed by Dr. Azucena García Palacios. Azucena García Palacios, where standard DBT was tested to treat Borderline Personality Disorder and Eating Behavior Disorders with good results in improving emotional dysregulation and impulsive behaviors in naturalistic settings in real clinical practice after treatment and in the long term (33,34) On the other hand, recent evidence has shown that the skills training component of DBT (where components of ER, mindfulness, distress tolerance, and interpersonal effectiveness are taught) alone is effective in reducing dysfunctional behaviors associated with emotional dysregulation and has been implemented as an intervention independent for several clinical conditions (35), including alcohol addiction (11,36). DBT has been

specifically adapted to treat Substance Use Disorders. This adaptation incorporates concepts and modalities designed to promote abstinence and reduce the duration and adverse impact of relapse (37). In studies conducted in Italy where only the group format was applied, low rates of alcohol relapse and low treatment dropout rates were obtained after the 3-month DBT skills training. Skills training has also shown preliminary support in reducing alcohol addiction severity, days of abstinence, as well as emotional dysregulation (11). In addition, change in ER difficulties during treatment was a mediator explaining treatment outcomes on primary (duration of abstinence maintenance (11,36) and secondary (experiential avoidance/emotional distress (11) variables.

In Spain, research on the efficacy of the skills training component of group therapy (compared to treatment as usual and medication) in treating emotional dysregulation is solid. Different randomized controlled studies have been conducted in patients with borderline personality disorder (38-40). However, to our knowledge, no data have been published on this intervention for treating addictions. There are somepreliminary results (presented at a congress) of this intervention in people with Borderline Personality Disorder and Substance Use Disorder (including alcohol) in outpatient treatment where improvements were found in impulsivity, emotional dysregulation and decreased alcohol use (41,42).

2. Unified Protocol:

In 2011, the first manual of the Unified Protocol for the transdiagnostic treatment of ETs (PU hereafter; (27) was published. Dr. David H. Barlow's team at Boston University developed this cognitive-behavioral treatment that directly addresses the shared mechanisms associated with neuroticism that cause the emotional dysregulation problem characteristic of all TEs. By focusing on shared mechanisms, PU can beused to treat a wide range of psychological disorders with a single intervention protocol, including presentations with comorbidity or unspecified symptoms. PU trains in adaptive ER skills from a learning, acceptance, and practice-based approach. The five core ER skills of PU are: mindfulness training, cognitive flexibility, identification/oppositional behavior of emotional behaviors, interoceptive exposure, and emotional exposure (27,43). We already have three systematic review and meta-analysis studies reporting the effectiveness of PU in improving the use of adaptive ER strategies (28-30). In Spain, our team has developed a line of research focused on the study of the cost-effectiveness, acceptability and implementation of PU in different formats (e.g., group) in the public health system. Our results show that PUachieves changes in both TE symptoms and transdiagnostic mechanisms, such as neuroticism, extraversion and difficulties in RE (44,45), the benefits are maintained up to 12 months from the end of treatment (46). We have also shown that PU in group format is cost-effective for our public system (47) and that clinical psychologists value it positively and would use it in the future, once the appropriate training has beencarried out (48). From the perspective of the PU, alcohol abuse would be understood as a maladaptive ER strategy; therefore, as in the case of TEs, emotional dysregulation would be understood as a strategy of maladaptive ER; therefore, as in the case of TEs, emotional dysregulation would be understood as a strategy of maladaptive ER.

may be a promising target for the improvement of treatments for people presenting comorbidity between alcohol addiction and one or more TEs (49). In this regard, PU has already demonstrated preliminary efficacy in decreasing alcohol abuse in patients with comorbidity with anxiety disorders through a double-blind, randomized, placebo-controlled study (50). The results suggest that a transdiagnostic treatment that addresses ER problems may be useful as a tool to manage alcohol abuse (51). Other studies have also shown its effectiveness in the treatment of other disorders with more severe ER difficulties such as bipolar disorder (52), people with self-injurious behavior (53) or borderline personality disorder (54).

Problems in the Dissemination and Implementation of Evidence-Based Psychological Treatments Because dissemination and implementation research is a relatively new area of study in the addictions field (55), little is known about how to optimize the implementation of evidence-based psychological treatments (EBPTs). In general, there is a major problem in translating efficacy studies of EBPTs into daily clinical practice. It is estimated that the time span from when a study is conducted until it is later implemented is 17-20 years (56,57). Because of this

In addition, a line of work has emerged through the implementation science that is developing

methods and procedures to ensure that EBSTs are more rapidly incorporated into daily clinical practice. Implementation science is the scientific study of methods to promote the incorporation of research findings and other evidence-based practices into daily practice, thereby improving the quality and efficiency of health services (58). One of the main barriers in the implementation of alcohol interventions is adequate training in EBT. The characteristics of the professionals are also important determinants of treatment adoption. With regard to facilitators, providers' familiarity with the interventions, perception of their effectiveness, and attitudes toward them have been found to be associated with the likelihood of successful implementation (59-61). Our research team has experience in implementation studies.

Regarding DBT, in research involving the PI of this project in the United States, 75% of mental health professionals (n=212) adopted all standard DBT treatment modes after receiving DBT training, and applied DBT to an average of 118 patients (62). Preliminary data presented to congress report higher rates (around 80%) of adoption by practitioners from Spain and Latin America after receiving DBT training (42). In addition, barriers to implementation have been investigated. The most commonly reported reasons in the studies regarding DBT program abandonment were those related to lack of organizational support such as lack of commitment from the administration, funding difficulties and insufficient resources (63,64). With reference to the PU, studies have been carried out on the acceptability and intention to use the PU in psychologists specializing in clinical psychology in the Spanish NHS (48), and general health psychologists (65), after receiving training in the PU. In both studies, high scores were obtained for overall acceptability (M = 4.30 outof 5, SD = 0.68, range = 3-5) and intention to use in the future (M = 4.54 out of 5, SD = 0.56, range = 3-5). Despite these promising results, it has been suggested that further research is needed to evaluate different variables that may influence the implementation process by professionals who have received DBT or PU training.

Research models on acceptance, intention to use and implementation of TPBEs.

The research methodology used in dissemination and implementation trials of EBTs differs from that of the efficacy and effectiveness trials that have traditionally been used. For example, unlike evaluating efficacy and effectiveness, research questions in dissemination and implementation focus on exploring the extent to which implementation of an intervention increases the speed of delivery, the quality of program delivery, and/or the quantity or degree of access or penetration of the intervention. While efficacy designs are based on randomized controlled trials in which

tests the intervention against control conditions/other interventions using rigid study protocols, implementation studies test the intervention(s) in the real context using flexible protocols customized to the specific needs of the service (66).

In order to frame the implementation strategies of an intervention, different models have also been developed that propose implementation variables to be investigated, among the best known, the RE-AIM (Reach, Effectiveness, Adoption, Implementation and Maintenance of the intervention, (67), the PARISH (Promoting Action on Research Implementation in Health Services, (68-70), or the CFIR (Consolidated Framework for Implementation Research, (71). Based on these models, Proctor et al. (72) proposed a series of key outcome variables to be evaluated in implementation studies. The most relevant and studied are: 1) acceptability: perception among implementation stakeholders that a particular treatment is pleasant, palatable or satisfactory; 2) adoption: intention, initial decision or action to try or employ an evidence-based practice; 3) appropriateness: perceived fit, relevance, compatibility, of the innovation to address a particular issue or problem; 4) feasibility: degree to which a new treatment can be used or carried out successfully in a given setting;

5) fidelity: degree to which an intervention has been carried out as prescribed in the original protocol or as intended by the program developers; 6) penetration: integration of a practice into a service setting; 7) sustainability: degree to which a newly implemented treatment is maintained or institutionalized within the stable and ongoing operations of a service setting. In this project, we will evaluate all the variables that this study will allow us to do, depending on the time allotted for this purpose.

variables will be evaluated in future studies.

In addition, implementation science proposes three main types of fundamental evaluation (58): 1) process evaluation: characteristics of the use of the intervention, measures before, during and after implementation); 2) formative evaluation: adding the above and providing feedback to the implementation team to adapt and improve the implementation process; 3) summative evaluation: carried out at the end of the study, assessing the impact of the intervention (increased use of the intervention in the target context oreconomic impact). In this research, taking into account the time span of the projects, we will focus on process and formative evaluations, where we will collect measures before, during and after the implementation process, providing feedback to the team through the supervisions and results of the focus groups (explained below) to adapt and improve the implementation process. For future studies, we will consider conducting a summative evaluation.

Justification of the study:
Alcohol use disorders (AUD) are very prevalent mental health disorders in the public health system and are associated with significant morbidity and mortality, as well as various comorbidities with other disorders that limit the effectiveness of psychological treatments. The scientific literature points to emotional dysregulation as one of the most important factors determining craving and relapse in people with alcohol addiction. In this sense, emotional dysregulation could represent a promising target for the improvement of psychological treatments for alcohol addiction. New proposals for transdiagnostic treatments provide a comprehensive working model in which substance use is considered as a means of coping with aversive emotional states. DBT and PU are two examples of transdiagnostic treatments with good results in different psychological disorders, and the group format of these interventions has shown good preliminary results in improving the severity of alcohol use. Research has traditionally focused on efficacy and effectivenessstudies of interventions. However, there is a major problem in translating the results of these studies into daily clinical practice. For this reason, implementation science has emerged, which is concerned with the scientific study of methods to promote the incorporation of research results and other evidence-based practices into daily practice to improve the quality and efficiency of health services. Dissemination and implementation research is a new and scarce area of study in the field of addictions. It has been found that one of the main barriers in the implementation of alcohol interventions is the lack of adequate training in evidence-based treatment. For all these reasons, we propose a dissemination, acceptability and implementation study that aims to train addiction professionals of the Spanish mental health system in two transdiagnostic treatments to treat emotional dysregulation, with the objective of promoting an adequate implementation of these interventions in the clinical practice of their work context. During the study, the effectiveness of dissemination and implementation strategies will be evaluated.

4.3 Bibliography (must be referenced in the preceding text)

1. American Psychiatric Association (2022). Diagnostic and statistical manual of mental disorders (5th ed., text rev.). https://doi.org/10.1176/appi.books.9780890425787
2. Connor JP, Haber PS, Hall WD. Alcohol use disorders. The Lancet. 2016;387(10022):988–98.
3. Schuckit MA. Alcohol-use disorders. The Lancet. 2009;373(9662):492–501.
4. World Health Organization. Global status report on alcohol and health [Internet]. 2022 [cited 2023 Jun 15]. Available from: https://www.who.int/es/news-room/fact-sheets/detail/alcohol
5. Spanish Observatory on Drugs and Addictions. Statistics. Alcohol, tobacco and illegal drugs in Spain. 2021 p. 213.
6. Technical Organization of Drug Addictions. Information system on drug dependence in Catalonia. 2008.
7. Bullis JR, Boettcher H, Sauer-Zavala S, Barlow DH. What is an emotional disorder ? A transdiagnostic mechanistic definition with implications for assessment , treatment , and prevention. Clin Psychol Sci Pract. 2019;1-19.
8. Santos-de Pascual A, Saura-Garre P, López-Soler C. Mental health in persons with Parkinson's disease. Substance use: differential aspects between men and women. An Psicol. 2020; 36(3):443- 50.

9. Gamble SA, Conner KR, Talbot NL, Yu Q, Tu XM, Connors GJ. Effects of Pretreatment and Posttreatment Depressive Symptoms on Alcohol Consumption Following Treatment in Project MATCH. J Stud Alcohol Drugs. 2010 Jan;71(1):71-7.
10. Sinha R. How does stress lead to risk of alcohol relapse? Alcohol Res Curr Rev. 2012;34(4):432.
11. Cavicchioli M, Movalli M, Vassena G, Ramella P, Prudenziati F, Maffei C. The therapeutic role of emotion regulation and coping strategies during a stand-alone DBT Skills training program for alcohol use disorder and concurrent substance use disorders. Addict Behav. 2019 Nov;98:106035.
12. Petit G, Luminet O, Maurage F, Tecco J, Lechantre S, Ferauge M, et al. Emotion Regulation in Alcohol Dependence. Alcohol Clin Exp Res. 2015 Dec;39(12):2471-9.
13. Jackson KM, Sher KJ. Alcohol use disorders and psychological distress: a prospective state-trait analysis. J Abnorm Psychol. 2003;112(4):599.
14. Conway KP, Swendsen JD, Rounsaville BJ, Merikangas KR. Personality, drug of choice, and comorbid psychopathology among substance abusers. Drug Alcohol Depend. 2002;65(3):225-34.
15. Anker J, Kushner M, Thuras P, Menk J, Unruh A. Drinking to cope with negative emotions moderates alcohol use disorder treatment response in patients with co-occurring anxiety disorder. Drug Alcohol Depend. 2016;159:93-100.
16. Ritschel LA, Lim NE, Stewart LM. Transdiagnostic applications of DBT for adolescents and adults. Am J Psychother. 2015; 69(2):111-28.
17. Aldao A, Nolen-Hoeksema S. When are adaptive strategies most predictive of psychopathology? J Abnorm Psychol. 2012; 121(1):276-81.
18. Kring AM, Sloan DM. Emotion regulation and psychopathology: A transdiagnostic approach to etiology and treatment. Guilford Press; 2009.
19. Norton PJ, Paulus DJ. Toward a unified treatment for emotional disorders: update on the science and practice. Behav Ther. 2016; 47(6):854-68.
20. Fernandez KC, Jazaieri H, Gross JJ. Emotion Regulation: A Transdiagnostic Perspective on a New RDoC Domain. Cogn Ther Res. 2016 Jun;40(3):426-40.
21. Aldao A, Nolen-Hoeksema S, Schweizer S. Emotion-regulation strategies across psychopathology: A meta-analytic review. Clin Psychol Rev. 2010 Mar;30(2):217-37.
22. Aldao A, Nolen-Hoeksema S. The influence of context on the implementation of adaptive emotion regulation strategies. Behav Res Ther. 2012;50(7-8):493-501.
23. McLaughlin KA, Nolen-Hoeksema S. Rumination as a transdiagnostic factor in depression and anxiety. Behav Res Ther. 2011;49(3):186-93.
24. Newby JM, McKinnon A, Kuyken W, Gilbody S, Dalgleish T. Systematic review and meta-analysis of transdiagnostic psychological treatments for anxiety and depressive disorders in adulthood. Clin Psychol Rev. 2015 Aug;40:91-110.
25. Linehan MM. Skills training manual for treating borderline personality disorder. Guilford press; 1993.
26. Linehan MM, Wilks CR. The course and evoluϴon of dialectical behavior therapy. Am J Psychother. 2015;69(2):97-110.
27. Barlow DH, Farchione TJ, Fairholme CP, Ellard KK, Boisseau CL, Allen LB, et al. The Unified Protocol for Transdiagnostic Treatment of Emotional Disorders. New York: Oxford University Press; 2011.
28. Carlucci L, Saggino A, Balsamo M. On the efficacy of the unified protocol for transdiagnostic treatment of emotional disorders: A systematic review and meta-analysis. Clin Psychol Rev. 2021 Jul;87:101999.
29. Sakiris N, Berle D. A systematic review and meta-analysis of the Unified Protocol as a transdiagnostic emotion regulation based intervention. Clin Psychol Rev. 2019 Aug;72:101751.
30. Cassiello-Robbins C, Southward MW, Tirpak JW, Sauer-Zavala S. A systematic review of Unified Protocol applications with adult populations: Facilitating widespread dissemination via adaptability. Clin Psychol Rev. 2020 Apr;101852.
31. Valentine SE, Smith AM, Stewart K. A review of the empirical evidence for DBT skills training as a stand-alone intervention. Handb Dialect Behav Ther. 2020;325-58.
32. Neacsiu AD, Bohus M, Linehan MM. Dialectical behavior therapy: An intervention for emotion dysregulation. Handb Emot Regul. 2014;2:491-507.
33. Navarro-Haro MV, Botella C, Guillen V, Moliner R, Marco H, Jorquera M, et al. Dialectical behavior therapy in the treatment of borderline personality disorder and eating disorders comorbidity: A pilot study in a naturalistic seţng. Cogn Ther Res. 2018;42:636-49.

34. Navarro-Haro MV, Botella VG, Badenes-Ribera L, Borao L, García-Palacios A. Dialectical Behavior Therapy in the Treatment of Comorbid Borderline Personality Disorder and Eating Disorder in a Naturalistic Seţng: A Six-Year Follow-up Study. Cogn Ther Res. 2021;45:480-93.
35. Valentine SE, Bankoff SM, Poulin RM, Reidler EB, Pantalone DW. The use of dialectical behavior therapy skills training as stand-alone treatment: A systematic review of the treatment outcome literature. J Clin Psychol. 2015;71(1):1-20.
36. Maffei C, Cavicchioli M, Movalli M, Cavallaro R, Fossati A. Dialectical behavior therapy skills training in alcohol dependence treatment: findings based on an open trial. Subst Use Misuse. 2018;53(14):2368-85.
37. Dimeff LA, Linehan MM. Dialectical behavior therapy for substance abusers. Addict Sci Clin Pract. 2008;4(2):39.
38. Feliu-Soler A, Pascual JC, Elices M, Marơn-Blanco A, Carmona C, Cebolla A, et al. Fostering self compassion and loving-kindness in patients with borderline personality disorder: A randomized pilot study. Clin Psychol Psychother. 2017;24(1):278-86.
39. Soler J, Pascual JC, Campins J, Barrachina J, Puigdemont D, Alvarez E, et al. Double-blind, placebo controlled study of dialectical behavior therapy plus olanzapine for borderline personality disorder. Am J Psychiatry. 2005;162(6):1221-4.
40. Soler J, Pascual JC, Tiana T, Cebrià A, Barrachina J, Campins MJ, et al. Dialectical behaviour therapy skills training compared to standard group therapy in borderline personality disorder: a 3-month randomized controlled clinical trial. Behav Res Ther. 2009;47(5):353-8.
41. Navarro Haro M. DBT-SUD to treat TUS: preliminary data. Online format presented at; 2021 Mar 26; Conference for mental health professionals on DBT.
42. Mercedes Jorquera, María Vicenta Navarro Haro, Pablo Calvo, Francesca Rubio, Cristina Borra, Isabel; Fernández Felipe, Lorién Ara, Azucena García Palacios. Dialectical Behavior Therapy to treat Borderline Personality Disorder and Substance Use Disorder: Preliminary Data. 2022 Sep 27; Second Iberoamerican Congress of DBT.
43. Barlow DH, Farchione TJ, Sauer-Zavala S, Latin HM, Ellard KK, Bullis JR, et al. Unified protocol for transdiagnostic treatment of emotional disorders: Therapist guide. New York, NY: Oxford University Press; 2018.
44. Osma J, Peris-Baquero O, Suso-Ribera C, Sauer-Zavala S, Barlow DH. Predicting and Moderating the Response to the Unified Protocol: Do Baseline Personality and Affective Profiles Matter? Cogn Ther Res. 2021;
45. Peris-Baquero Ó, Moreno-Pérez JD, Navarro-Haro MV, Díaz-García A, Osma J. Emotion dysregulation and neuroticism as moderators of group Unified Protocol effectiveness outcomes for treating emotional disorders. J Affect Disord. 2023 Jun;331:313-21.
46. Peris-Baquero O, Osma J. Unified Protocol for the Transdiagnostic Treatment of Emotional Disorders in Group Format in Spain: Results of a Noninferiority Randomized Controlled Trial at 15 Months aŌer Treatment Onset. Landi G, editor. Depress Anxiety. 2023 Jun 30;2023:1-16.
47. Peris-Baquero Ó, Moreno JD, Osma J. Long-term cost-effectiveness of group unified protocol in the Spanish public mental health system. Curr Psychol [Internet]. 2022 Jun 24; Available from: https://link.springer.com/10.1007/s12144-022-03365-8
48. Peris-Baquero Ó, Osma J, Gil-LaCruz M, Marơnez-García L. Acceptability of and intention to use the Unified Protocol delivered in group format in the Spanish Public Health System. J Eval Clin Pract. 2021 Feb 9;jep.13546.
49. Farchione TJ, Fitzgerald HE, Curreri A, Janes AC, Gallagher MW, Sbi S, et al. Efficacy of the Unified Protocol for the treatment of comorbid alcohol use and anxiety disorders: Study protocol and methods. Contemp Clin Trials. 2021 Sep;108:106512.
50. Ciraulo DA, Barlow DH, Gulliver SB, Farchione T, Morissette SB, Kamholz BW, et al. The effects of venlafaxine and cognitive behavioral therapy alone and combined in the treatment of co-morbid alcohol use-anxiety disorders. Behav Res Ther. 2013;51(11):729-35.
51. Landa González N, Osma J, Peris-Baquero O. Emotional disorders and comorbidity with addictions. In: Applications of the Unified Protocol for the transdiagnostic treatment of dysregulation. emotional. Madrid: Alianza Editorial; 2019.

52. Ellard KK, Bernstein EE, Hearing C, Baek JH, Sylvia LG, Nierenberg AA, et al. Transdiagnostic treatment of bipolar disorder and comorbid anxiety using the Unified Protocol for Emotional Disorders: A pilot feasibility and acceptability trial. J Affect Disord. 2017 Sep;219:209-21.
53. Bentley KH. Applying the Unified Protocol Transdiagnostic Treatment to Nonsuicidal Self-Injury and Co-Occurring Emotional Disorders: A Case Illustration. J Clin Psychol. 2017;73(5).
54. Lopez ME, Stoddard JA, Noorollah A, Zerbi G, Payne LA, Hitchcock CA, et al. Examining the Efficacy of the Unified Protocol for Transdiagnostic Treatment of Emotional Disorders in the Treatment of Individuals With Borderline Personality Disorder. Cogn Behav Pract. 2015 Nov;22(4):522-33.
55. Gold PB, Glynn SM, Mueser KT. Challenges to Implementing and Sustaining Comprehensive Mental Health Service Programs. Eval Health Prof. 2006 Jun;29(2):195-218.
56. Green B. Understanding and Researching Professional Practice. 2009; 5.
57. Morris ZS, Wooding S, Grant J. The answer is 17 years, what is the question: understanding time lags in translational research. J R Soc Med. 2011;104(12):510-20.
58. Bauer MS, Damschroder L, Hagedorn H, Smith J, Kilbourne AM. An introduction to implementation science for the non-specialist. BMC Psychol. 2015 Dec 16;3(1):32.
59. Obert JL, Brown AH, Zweben J, Christian D, Delmhorst J, Minsky S, et al. When treatment meets research: Clinical perspectives from the CSAT Methamphetamine Treatment Project. J Subst Abuse Treat. 2005 Apr;28(3):231-7.
60. Herbeck DM, Hser YI, Teruya C. Empirically supported substance abuse treatment approaches: A survey of treatment providers' perspectives and practices. Addict Behav. 2008 May;33(5):699-712.
61. Thomas CP, Wallack SS, Lee S, McCarty D, Swio, R. Research to practice. J Subst Abuse Treat. 2003 Jan;24(1):1-11.
62. Navarro-Haro MV, Harned MS, Korslund KE, DuBose A, Chen T, Ivanoff A, et al. Predictors of adoption and reach following dialectical behavior therapy intensive trainingTM. Community Ment Health J. 2019;55:100-11.
63. Carmel A, Fruzzet, AE, Rose ML. Dialectical Behavior Therapy Training to Reduce Clinical Burnout in a Public Behavioral Health System. Community Ment Health J. 2014 Jan;50(1):25-30.
64. Swales MA, Taylor B, Hibbs RAB. Implementing Dialectical Behaviour Therapy: Programme survival in routine healthcare settings. J Ment Health. 2012 Dec;21(6):548-55.
65. Peris-Barquero Ó, Ósma JJ, Maronez-Segura V. Study on the use, acceptability and implementation of the unified protocol in general health psychologists in Spain. An Psicol Psychol. 2022;38(3):399- 408.
66. Landsverk J, Brown CH, Smith JD, Chamberlain P, Curran GM, Palinkas L, et al. Design and Analysis in Dissemination and Implementation Research [Internet]. Vol. 1. Oxford University Press; 2017 [cited 2023 Jul 5]. Available from: https://academic.oup.com/book/26456/chapter/194882719
67. Glasgow RE, Vogt TM, Boles SM. Evaluating the public health impact of health promotion interventions: the RE-AIM framework. Am J Public Health. 1999 Sep;89(9):1322-7.
68. Kitson A, Harvey G, McCormack B. Enabling the implementation of evidence based practice: a conceptual framework. BMJ Qual Saf. 1998;7(3):149-58.
69. Rycroff-Malone J, Gill H, Kitson A. Getting evidence into practice: ingredients for change. Nurs Stand 2013. 2002;16(37):38.
70. Rycroff-Malone J. Evidence-informed practice: from individual to context. J Nurs Manag. 2008;16(4):404-8.
71. Damschroder LJ, Reardon CM, Widerquist MAO, Lowery J. The updated Consolidated Framework for Implementation Research based on user feedback. Implement Sci. 2022 Oct 29;17(1):75.
72. Proctor E, Silmere H, Raghavan R, Hovmand P, Aarons G, Bunger A, et al. Outcomes for implementation research: conceptual distinctions, measurement challenges, and research agenda. Adm Policy Ment Health Ment Health Serv Res. 2011;38:65-76.
73. Teddlie C, Tashakkori A. Foundations of mixed methods research: Integrating quantitative and qualitative approaches in the social and behavioral sciences. Sage; 2009.
74. Barlow DH, Farchione TJ, Bullis JR, Gallagher MW, Murray-Latin H, Sauer-Zavala S, et al. The Unified Protocol for Transdiagnostic Treatment of Emotional Disorders Compared With Diagnosis-Specific Protocols for Anxiety Disorders. JAMA Psychiatry. 2017 Sep;74(9):875.

75. Aarons GA. Mental Health Provider Attitudes Toward Adoption of Evidence-Based Practice: The Evidence-Based PracƟce AƩtude Scale (EBPAS). Ment Health Serv Res. 2004 Jun;6(2):61-74.
76. Shea CM, Jacobs SR, Esserman DA, Bruce K, Weiner BJ. Organizational readiness for implementing change: a psychometric assessment of a new measure. Implement Sci. 2014;9(1):1-15.
77. Sekhon M, Cartwright M, Francis JJ. Acceptability of healthcare interventions: an overview or reviews and development of a theoretical framework. BMC Health Serv Res. 2017 Dec 26;17(1):88-
78. Lavrakas PJ. Encyclopedia of Survey Research Methods. SAGE Publications; 2008.
79. Bryman A. Social research methods. Oxford university press; 2016.
80. Finch T, Girling M, May C, Mair F, Murray E, Treweek S, et al. NoMAD: implementation measure based on normalization process theory. Meas Instrum. 2015.
81. Hennink M, Kaiser B, Weber M. What Influences Saturation? Estimating Sample Sizes in Focus Group Research. Qual Health Res. 2019;29(10):1483-96.
82. Ruiz-Rodríguez P, Cano-Vindel A, Navarro RM, Medrano L, Moriana JA, Aguado CB, et al. Economic impact and burden of common mental disorders in Spain: A systematic and critical review. Anxiety Stress. 2017;23(2-3):118-23.
83. Osma J, Suso-Ribera C, García-Palacios A, Crespo-Delgado E, Robert-Flor C, Sánchez-Guerrero A, et al. Efficacy of the unified protocol for the treatment of emotional disorders in the Spanish public mental health system using a group format: Study protocol for a multicenter, randomized, non-inferiority controlled trial. Health Qual Life Outcomes. 2018;16(1):46.
84. IBM Corp. IBM SPSS Statistics for Windows, Version 25.0. Armonk, NY: IBM Corp; 2017.
85. Kuckartz U, Rädiker S. Analyzing qualitative data with MAXQDA. Springer; 2019.
86. Schreier M. Qualitative content analysis in practice. Sage publications; 2012.
87. Ehrhart MG, Aarons GA, Farahnak LR. Assessing the organizational context for EBP implementation: the development and validity testing of the Implementation Climate Scale (ICS). Implement Sci. 2014;9:157.
88. Ehrhart, M.G., Torres, E.M., Hwang, J. *et al.* Validation of the Implementation Climate Scale (ICS) in substance use disorder treatment organizations. *Subst Abuse Treat Prev Policy* 14, 35 (2019). https://doi.org/10.1186/s13011-019-0222-5
89. Kristensen TS, Borritz M, Villadsen E, Christensen KB. The Copenhagen Burnout Inventory: a new tool for the assessment of burnout. Work & Stress. 2005; 19: 192-207.
90. Molinero Ruiz E, Basart Gómez-Quintero H, Moncada Lluis S. Validation of the Copenhagen Burnout Inventory to assess professional burnout in Spain. Rev Esp Public Health. 2013 Mar-Apr;87(2):165- 79. English, Spanish. doi: 10.4321/S1135-57272013000200006.
91. Humphreys, K., Greenbaum, M. A., Noke, J. M., & Finney, J. W. (1996). Reliability, validity, and normative data for a short version of the Understanding of Alcoholism Scale. *Psychology of Addictive Behaviors, 10*(1), 38-44. https://doi.org/10.1037/0893-164X.10.1.38.
92. Sauer-Zavala, S., Ametaj, A. A., Wilner, J. G., Bentley, K. H., Marquez, S., Patrick, K. A., Starks, B., Shtasel, D., & Marques, L. (2019). Evaluating transdiagnostic, evidence-based mental health care in a safety-net setting serving homeless individuals. *Psychotherapy, 56*(1), 100-114. https://doi.org/10.1037/pst0000187.
93. Hawkins KA, Sinha R. Can line clinicians master the conceptual complexities of dialectical behavior therapy? An evaluation of a state department of mental health training program. J Psychiatr Res. 1998. https://doi.org/10.1016/S0022-3956(98)00030-2.
94. Dimeff LA, Koerner K, Woodcock EA, Beadnell B, Brown MZ, Skutch JM, et al. Which training method words best? A randomized controlled trial comparing three methods of training clinicians in dialectical behavior therapy skills. Behav Res Ther. 2009. https://doi.org/10.1016/j.brat.2009.07.011.
95. Luke DA, Calhoun A, Robichaux CB, Elliott MB, Moreland-Russell S. The Program Sustainability Assessment Tool: A New Instrument for Public Health Programs. Prev Chronic Dis 2014;11:130184. DOI: http://dx.doi.org/10.5888/pcd11.130184
96. Weiner, B. J., Lewis, C. C., Stanick, C., Powell, B. J., Dorsey, C. N., Clary, A. S., Boynton, M. H., and Halko, H. (2017). Psychometric assessment of three newly developed implementation outcome measures. Implementation Science, 12(108), 1-12. doi: 10.1186/s13012-017-0635-3.

4.4 Hypothesis (assertion to be proved)

Study 1: Attitudes, acceptability and intention to use the interventions and adaptation of the interventions to the real context where they will be applied (pre-implementation):
H1. There will be a significant increase in positive attitudes toward EBTs by practitioners about DBT and PU treatments after each training.

H2. Organizational readiness for treatment implementation will increase significantly after each training by the professionals.

H3. Acceptability and intention to use the interventions after each training will be rated positively in both interventions.

H4. Throughout the training and at the end of it, the professionals, based on their clinical experience, will provide relevant information for the adaptation of both interventions to the real context where they will be applied.

Study 2: Implementation of Transdiagnostic Interventions (DBT and PU)

H1. Good results of appropriateness, adoption, reach and maintenance of DBT and PU in clinical practice will be found after training.

H2. Based on previous studies mentioned above, the main barriers could be related to the lack of administrative support and economic resources, as well as the lack of reduction of the burden of clinician responsibilities that would be necessary to implement the interventions. With respect to facilitators, they would be related to provider familiarity with the interventions, the perception of their effectiveness and positive attitudes towards them.

H3. The predictor variables of successful implementation will be related to the characteristics of the professionals and the organization.

4.5 Objectives

Two studies are proposed, the second being the natural continuation of the first.

Study 1: Attitudes, acceptability and intention to use the interventions and adaptation of the interventions to the real context where they will be applied (pre-implementation).

General objective: To assess the effect of dissemination on attitudes, readiness to change, acceptability, and intention to use two transdiagnostic treatments (DBT and PU) in mental health professionals treating people with alcohol addiction, as well as to explore information relevant to the adaptation and appropriateness of both interventions for use in real-world settings.

Specific objectives:
O1. Determine attitudes toward EBPTs of mental health professionals about DBT and PU treatments before and after each training.

O2. To assess the organizational readiness for the implementation of DBT and PU treatments before and after each training by mental health professionals.

O3. To explore factors related to acceptability and intention to use (attitude, perceived efficacy, intervention consistency, self-efficacy, overall acceptability and intention to use) of the interventions after each training (DBT and PU).

O4. To gather the opinions, advice and recommendations of mental health professionals working with people with alcohol addiction on the adaptations that should be made to their work.

be made so that the interventions (DBT and PU) can be used in the natural/actual context.

Study 2: Implementation of DBT and PU Interventions
General objective: To evaluate the process of implementation of emotional regulation interventions (DBT and PU) by the professionals who decide to implement the interventions after the training.

Specific objectives:
O1. Evaluate implementation outcomes (adoption, reach, appropriateness, feasibility, fidelity, sustainability) of DBT and PU in clinical practice by practitioners who choose to implement interventions after training.
O2. Determine the number and type of barriers and facilitators during the process of implementing DBT and PU in clinical practice by professionals who decide to implement the interventions after training.

O3. To explore the predictor variables of a successful implementation taking into account the previous characteristics of the professionals and the organization.

4.6 Methodology (<u>**all of**</u> the following fields must be detailed**):**
Study design
<u>Participants</u>: inclusion/exclusion criteria; mode of recruitment (who makes initial contact with participants and how, present study dissemination material, if any), sample size (and justification), randomization (if applicable).
<u>Sources of information</u>: detailed variables (data to be collected), origin of the data, when and how it is collected, to what period of time it refers.
<u>Procedures</u>: detail in a differentiated way the purely assistance procedures from those of the research, present surveys or forms to be used (link in case of online surveys), risk assessment of the experimental procedures and measures to minimize it.
<u>Statistical analysis Limitations</u>
<u>of the study</u>
<u>In case of biological samples</u>: detail type and number of samples, how they are collected, where and by whom they are analyzed, when they are destroyed (or final destination).

<u>Design:</u>
Parallel Mixed-Methods-Design will be used for this project. This type of design is used when quantitative and qualitative research data are combined. In the case of "parallel" mixed designs, there are two or more parallel quantitative and qualitative data streams, either with some minimum time span or simultaneously. The results of these investigations are integrated in meta-inferences after separate analyses (73).

<u>Participants:</u>

<u>Study 1:</u>
Participants:
Mental health professionals (psychiatrist, psychologist and nurse) who work in the Drug Addiction Care Resources of the NHS in Aragon, Valencia and Catalonia and are treating users with alcohol addiction will be selected.

Inclusion Criteria: 1) Be at least 18 years of age; 2) Mental health professional currently working in a drug dependence care resource; 3) Agree to receive training in DBT and PU; 4) Understand Spanish language, 5) Accept informed consent.

Exclusion criteria: 1) No interest in receiving training in emotional regulation interventions;
2) Not including in its functions the psychological treatment of people with alcohol addiction; 3) Not having an Internet connection to be able to connect to the training sessions.

Since the trainings will be conducted sequentially and the evaluation of the trainings could be influenced by primacy and recency effects, we will randomize participants to receive DBT or PU training first, whereby half of the participants will receive DBT first and the other half PU.

Sample size: Taking into account the statistical analyses proposed, through the G*Power software, a total sample size of 160 participants was obtained for study 1 with a statistical power of 95%, an alpha coefficient of 0.05 and a conservative effect size of 0.25. On the other hand, to calculate the sample size, the number of professionals working in addiction resources that could collaborate in each community will also be considered (Information in appendix).

Study 2:
Participants: Mental health professionals (psychiatrist, psychologist and nurse) working in the Drug Addiction Care Resources of Aragon, Valencian Community and Catalonia and treating users with alcohol addiction and who have received the training in study 1.

Inclusion Criteria: 1) Be at least 18 years of age; 2) Mental health professional currently working in a mental health care setting; 3) Be at least 18 years of age; 4) Be at least 18 years of age.

3) To have received training in DBT and PU interventions in study 1; 4) To accept the implementation of one of the 2 interventions and supervision during the same; 5) To understand the Spanish language; 6) To accept informed consent.

Exclusion criteria: 1) No interest in receiving supervision in emotional regulation interventions;
2) Not including in its functions the psychological treatment of people with alcohol addiction; 3) Not having an Internet connection to be able to connect to the supervision sessions.

Since practitioners will be trained in both interventions, it is expected that they will be able to apply both in their context in order to evaluate the implementation process. Therefore, we will randomize participants to implement either DBT or PU, whereby half of the participants will first implement DBT and the other half PU, and then the next intervention. Due to the limited time of the study, only the first of the implemented interventions will be evaluated.

Sample size: In order to guarantee the implementation of the interventions in emotional regulation, supervision will be carried out session by session with the aim of analyzing the fidelity to the treatment and the implementation process in at least 20% of the professionals (n=32) who have received the training and have shown interest in participating in this second study. The choice of this percentage of participants, similar to those carried out in other studies where this type of intervention was used, will allow us to obtain a representative sample.

<u>Sources of information</u>:

Study 1: Attitudes, acceptability and intention to use the interventions and adaptation of the interventions to the real context where they will be applied (pre-implementation).

Pre- and post-training measurements:

Quantitative measures:

1. Characteristics of the professionals:

- Sociodemographic and labor questionnaire: designed by the team to collect demographic information (age, sex, educational level, marital status, employment status), information on the type of discipline exercised, theoretical orientation and population treated, as well as experience in DBT and PU and level of training (hours of previous training in emotional regulation). To be applied prior to training.

2. Attitudes toward evidence-based practice:

-Evidence Based Practice Attitude Scale (EBPAS; 75). This 15-item scale assesses participants' attitudes toward adopting evidence-based practice in four domains: 1) likelihood of adopting EBP given the requirements to do so, 2) intuitive appeal of EBP, 3) openness to new practices, and 4) perceived divergence of usual practice from EBP. The tool presents an overall scale alpha range of .77-.79 and an α-range of .78-.
0.93 for the subscales, excluding perceived divergence with somewhat lower reliability: α-range of .59 -
.66. To be applied before and after training.

3. Organizational willingness to implement evidence-based treatments:

-Organizational Readiness for Implementing Change (ORIC; 76). The ORIC is composed of 12 items divided into two main subscales: 1) 6 items on commitment to change (i.e., do the intended members of the organization want change?) and 2) 6 items on effectiveness of change (i.e., can the members of the organization change?). Cronbach's alpha values were, respectively, 0.91 and 0.89 for the Commitment to Change Scale and the Change Effectiveness Scale. This questionnaire has been translated into Spanish by ImpleMentAll partners (https://www.implementall.eu/9-outcomes-and-resources.html#ORICtranslations). It will be applied before and after the training.

4. Acceptability and intended use:
- Acceptability and Intention to Use Survey (48). This instrument is based on the Theoretical Framework of Acceptability (TFA), developed by Sekhon et al. (2017) that assesses acceptability and unifies approaches into a single theoretical framework. The model is composed of seven constructs: (1) Affective attitude; (2) Burden; (3) Ethics;
(4) Consistency of the intervention); (5) Opportunity costs; (6) Perceived efficacy; (7) Self-efficacy. This questionnaire was created ad hoc by the research team and also adds two more items reflecting general acceptability and intention to use the intervention in the future. It will be applied before and after the training.

- Acceptability Measure of the Intervention (AIM), Intervention Appropriateness Measure (IAM) and Feasibility of the intervention (96). This is an instrument with three scales (one for each variable), with four items each containing statements about the intervention to be measured. It presents Likert-type responses, where 1 is completely disagree and 5 is completely agree. This instrument presents solid psychometric properties: the Cronbach's alphas were between .87 and .89 for the total scale and by subscales the alphas were .85 for acceptability, .91 for appropriateness and .89 for feasibility (Weiner et al., 2017).

5. Climate of implementation:
-Implementation Climate Scale (ICS;87). This 18-item measure assesses the degree to which there is a strategic organizational climate that supports the implementation of evidence-based practices. Implementation climate is defined as the perception of the policies, practices, procedures, and behaviors thatare rewarded, supported, and expected to facilitate the effective implementation of EBP. We will use the version validated for substance use disorder treatment organizations (88). Cronbach's alpha reliabilities for the subscales and the ICS total score ranged from 0.78 to 0.90. This scale will be administered before and after the trainings.

6. Emotional state of the professionals:
-Copenhagen Burnout Inventory (CBI; 89). We will use the version validated for use in Spain and translated into Spanish (90). The questionnaire is in the public domain for the measurement of burnout syndrome, and is structured in three subdimensions: personal, work-related and work-related with clients. It presents good scores in the internal consistency of the three scales and a Cronbach's α of 0.90 in the personal, 0.83 in the work-related dimension and 0.82 in the dimension related to dealing with clients.

7. Attitudes towards the nature of alcohol consumption problems:
-Brief Scale of Understanding Substance Abuse (SUSS, 91): This instrument is composed of 19 items with statements about the nature and etiology of alcohol and substance abuse related disorders to which the evaluee must respond on a Likert scale from 0 (Strongly Disagree) to 4 (Strongly Agree) and consists of three subscales: disease model (7 items), psychosocial model (5 items) and eclectic orientation (7 items). This scale presents good scores for its first two scales (Cronbach's alphas of 0.86 and 0.72, respectively) and a low score of alpha=.61 in the eclectic orientation subscale.

8. Acquisition of therapists' knowledge of DBT and PU skills:
Tests that will assess the extent to which therapists have acquired knowledge about both therapeutic approaches will be developed ad hoc for this study.
-The knowledge acquisition test on the Unified Protocol will be based on the questionnaire of a study carried out by the authors for the same purpose (92) and which they provide in the annex to the publication of their article. It will consist of a series of multiple-choice questions on the contents of the PU training, from which the correct option will be selected and the number of correct and incorrect answers will be counted.
-The DBT skills knowledge acquisition test will be conducted taking into account the information collected in the following studies (93, 94) in which this variable was also assessed. It will consist of a series of multiple-choice questions on the contents of DBT-SUD training among which.

will have to select the correct option and the number of hits and misses will be counted.

Qualitative measures:
8. Focus group interview: Focus groups consist of group interviews with a small group of participants. These interviews will be recorded preserving anonymity. The objective is to allow participants to express their opinions, thoughts and experiences in relation to a specific topic (78). The methodology consists of developing an interview script that will include questions of interest to cover the general objective of the project, as well as specific study questions (79). In this case, questions will be included that will allow to know the acceptability and intention to use both interventions (DBT and PU), as well as the adaptations that would be necessary to implement them in clinical practice. It will be carried out after receiving both trainings.

Study 2:
Pre- and post-training measurements:

Quantitative and qualitative measures:
1. Characteristics of the professionals:
- Sociodemographic and labor questionnaire: designed by the team to collect demographic information (age, sex, educational level, marital status, employment status), information on the type of discipline exercised, theoretical orientation and population treated, as well as experience in DBT and PU and level of training (hours of previous training in emotional regulation). To be applied prior to training.

2. Adequacy of evidence-based treatments:
- Normalization Measure Development Questionnaire (NoMAD; 80). Questionnaire that measures implementation processes from the perspective of professionals directly involved in the implementation of complex interventions in health care. It has 20 items that measure four dimensions: coherence of the intervention with daily routine, cognitive participation, collective action of individuals and groups to apply the innovation in daily practice, and reflective monitoring. We used the version translated into Spanish by the ImpleMentAll partners group (https://www.implementall.eu/9- outcomes-and-resources.html#NoMADtranslations). Most Cronbach's alphas values for the four subscales reached a satisfactory internal consistency ($\alpha \geq 0.70$). To be applied before and after the implementation of the interventions.

3. Fidelity of the intervention:
-To evaluate the fidelity of the interventions in practice in real context, supervisors will use the standardized fidelity interviews used in "The DBT Adherence & Fidelity Project" (https://www.dbtadherence.com/) and the "Unified Protocol Institute" (http://www.unifiedprotocol.com) for supervision in DBT and PU. In these interviews, the degree of adequacy of the contents treated in the session with those foreseen by the manual, the degree of completion of the exercises in the session, the degree of achievement of the session objectives, the incidents and their resolution, the therapist's skill, patient adherence, or the perception of the therapeutic bond, among other variables, are evaluated.

4. Variables related to the implementation process:
- Focus group interview: Questions will be included to learn about some of the results of implementation, as well as the barriers and facilitators in the implementation of both interventions (DBT and PU). The following variables are initially proposed to be measured based on the proposals of Proctor et al.
(72): 1) acceptability; 2) adoption and scope; 3) appropriateness; 4) feasibility; 5) fidelity.

- Acceptability and Intention to Use Survey (48). This instrument is based on the Theoretical Framework of Acceptability (TFA), developed by Sekhon et al. (2017) that assesses acceptability and unifies approaches into a single theoretical framework. The model is composed of seven constructs: (1) Affective attitude; (2) Burden; (3) Ethics;
(4) Consistency of the intervention); (5) Opportunity costs; (6) Perceived efficacy; (7) Self-efficacy. This questionnaire was created ad hoc by the research team and also adds two more items reflecting general acceptability and intention to use the intervention in the future. It will be administered before and after the

training.

- Intervention Acceptability Measure (AIM), Intervention Appropriateness Measure (IAM) and Intervention Feasibility (96). This is an instrument with three scales (one for each variable), with four items each containing statements about the intervention to be measured. It presents Likert-type responses, where 1 is completely disagree and 5 is completely agree. This instrument has solid psychometric properties. Cronbach'salpha was between .87 and .89 and by subscales the alphas were .85 for acceptability, .91 for appropriateness and .89 for feasibility.

5. Organizational readiness:

-Organizational Readiness for Implementing Change (ORIC; 76). The ORIC is composed of 12 items divided into two main subscales: 1) 6 items on commitment to change (i.e., do the intended members of the organization want change?) and 2) 6 items on effectiveness of change (i.e., can the members of the organization change?). Cronbach's alpha values were, respectively, 0.91 and 0.89 for the Commitment to Change Scale and the Change Effectiveness Scale. It will be applied before and after the training. The version translated into Spanish by the ImpleMentAll partners group (https://www.implementall.eu/9-outcomes-and-resources.html#ORICtranslations) was used.

-Implementation Climate Scale (ICS;87). This 18-item measure assesses the degree to which there is a strategic organizational climate that supports the implementation of evidence-based practices. the implementation of evidence-based practices. The implementation climate is defined as the perception of the policies, practices, procedures, and behaviors that are rewarded, supported, and expected to facilitate the effective implementation of EBP. We will use the version validated for substance use disorder treatment organizations (88). Cronbach's alpha reliabilities for the subscales and the ICS total score ranged from 0.78 to 0.90. This scale will be administered before and after the trainings.

6. Implementation barriers:

-Barriers to Implementation Inventory (BTI; Behavioral Tech, LLC. (n.d.), unpublished instrument). The 39- item inventory consists of a list of barriers that teams may encounter when implementing DBT. The obstacles are structured by the following domains: team problems, administrative problems, theoretical/philosophical problems, and structural problems. The instrument presents a good internal consistency with a Cronbach's alpha .89. It will be applied after the implementation of the interventions.

6. Sustainability of the intervention:

-Program Sustainability Assessment Tool (PSAT, 95). This is a 40-item scale of intervention sustainability as reported by the organizational provider and stakeholders. Subscales include: political support, funding stability, stakeholder partnerships, organizational capacity, program evaluation, program adaptation, stakeholder communication, and strategic planning. The items are measured on a 7-point Likert scale (very little or no extent, very much). The mean internal consistency of the 8 subscales was .88 and ranged from.
.79 y .92. The Spanish version, developed by Washington University, St. Louis (USA), will be used (https://sustaintool.org/psat/assess/). This scale will be applied after the implementation of the interventions.

7. Emotional state of the professionals:

-Copenhagen Burnout Inventory (CBI; 89). We will use the version validated for use in Spain and translated into Spanish (90). The questionnaire is in the public domain for the measurement of burnout syndrome, and is structured in three subdimensions: personal, work-related and work-related with clients. It presents good scores in the internal consistency of the three scales and a Cronbach's α of 0.90 in the personal, 0.83 in the work-related dimension and 0.82 in the dimension related to dealing with clients.

8. Attitudes towards the nature of alcohol consumption problems:

-Brief Scale of Understanding Substance Abuse (SUSS, 91): This instrument is composed of 19 items with statements about the nature and etiology of alcohol and substance abuse related disorders to which the evaluee must respond on a Likert scale from 0 (Strongly Disagree) to 4 (Strongly Agree) and consists of three subscales: disease model (7 items), psychosocial model (5 items) and eclectic orientation (7 items). This scale presents good scores for its first two scales (Cronbach's alphas of 0.86 and 0.72, respectively), which are the ones that will be applied in the study. This instrument will be applied before and after the implementation of the interventions.

Procedures:

Study 1:

-Recruitment and invitation to participate: Recruitment of participants who meet the inclusion criteria for study 1 will be done by sending an email from the Mental Health and Addictions Service or Council responsible for each community. The agreements with the services responsible for disseminating the study information to the addiction resources in their community have been attached, where they will be contacted by email or telephone. In this institutional email, mental health professionals working in centers for people with alcohol addiction will receive information about the project, the professional and scientific team and theinvitation to participate in an informative meeting with the PI of the project. Once informed, the people who want to participate in the study will receive an email with a link to the information and informed consent form, and once they accept to participate, they will access the survey of a series of questionnaires that will becarried out online through the Qualtrics ® platform (https://www.qualtrics.com/es/). Participants will receive an invitation at each evaluation phase. Participants will be randomly assigned to receive DBT or PU training first [balanced randomization (1:1)], through the randomizer software, in order to eliminate possible biases and ensure equivalence between groups.

- Development of the evaluation protocol: The quantitative data of the study will be collected through questionnaires validated in Spanish population that will be introduced in the online survey platform Qualtrics so that professionals can complete them telematically. Qualitative data will be collected through interviews with open-ended questions and through focus groups. The participants in the focus groups will be the professionals who give their consent to participate in the project. From the total sample, 6-8 participants will be randomly selected to be part of the first focus group (random sampling). Participants will be selected randomly and groups of 6-8 people will continue to be carried out until saturation of the discourse is reached, that is, when no new information emerges after the analysis of the qualitative information. As indicated in the literature, we expect to reach discourse saturation after having conducted and analyzed about three focus groups, or in other words, after having included approximately 20-30 participants in the focus groups (81). For the collection and recording of the focus groups, the Zoom platform will be used. The privacy policy is described here: https://explore.zoom.us/es/gdpr/

- Evaluation phases:

The following table summarizes the evaluation measures in each evaluation phase.

Evaluation phase	Quantitative measurements	Qualitative measurements
Before each training (DBT and PU)	- Sociodemographic and labor questionnaire<br>- Evidence-Based Practice Attitudes Scale (EBPAS).<br>- Implementation Climate Scale (ICS)	

	- Burnout Questionnaire (CBI)<br>- Brief Understanding Substance Abuse Scale (SUSS)	
After each training (DBT and PU)	- Acceptability and intention to use survey (Peris-Baquero et al., 2021).<br>- Evidence-Based Practice Attitudes Scale (EBPAS).<br>- Organizational Readiness for Change Implementation (ORIC)<br>- Implementation Climate Scale (ICS)<br>- Burnout Questionnaire (CBI)<br>- Brief Understanding Substance Abuse Scale (SUSS)<br>- Acceptability Measures (AIM), Opportunity (IAM) and Feasibility (FIM) of the Intervention<br>- Knowledge acquisition test on PU and DBT of therapists.	- Focus group on acceptability and intention of use and adaptation of interventions<br>-

- DBT and PU training:

The training will consist of a 20-hour workshop in online format with the following contents:
1. DBT training:

Block 1
1.1. Introduction to key concepts of DBT: acceptance and change techniques, modes of treatment, treatment goals, dialectical philosophy, etiological and maintenance model to explain emotional dysregulation.
1.2. DBT-SUD Program (dialectical abstinence model, attachment strategies, behavioral analysis)
1.3. Why DBT for the treatment of alcohol addiction?
1.4. Main characteristics of DBT. Content distribution proposals for the group intervention format.

Block 2
2.1. DBT Skills Training to treat addiction: Mindfulness Skills
Discomfort tolerance skills
Skills for dealing with addiction Emotional regulation skills

Interpersonal effectiveness skills Self-
management and rehabilitation skills

2. PU training:
Block 1
Introduction. Theoretical justification of the transdiagnostic approach with special emphasis on its definition, the theoretical model on the etiology and maintenance of emotional disorders (triple vulnerability model), the definition of the identified transdiagnostic dimensions, the difficulties in emotional regulation following James Gross' model and its clinical implications. 1.2.Why PU for the treatment of alcohol addiction?
1.3. Dimensional evaluation of TEs and formulation of cases according to the PU
1.4. Main characteristics of the PU. Content distribution proposals for the group intervention format.

Block 2
2.1. Unified Protocol Modules: session by session:
M1. Setting goals and maintaining motivation M2.
Understanding emotions
M3. Full emotional awareness M4.
Cognitive Flexibility
M5. Opposing emotional behaviors M6.
Understanding and coping with physical
sensations M7. Emotional exposures
M8. Recognize your achievements and look to the future

Study 2
- Recruitment and invitation to participate:
The recruitment of participants who meet the inclusion criteria for study 2 will be carried out by sending an email from those responsible for each community. In this institutional email, the mental health professionals who have voluntarily participated in study 1 will receive information about study 2, the professional and scientific team that forms it and the invitation to participate from the PI of the project. Regarding the Qualtrics survey and the random assignment, the same procedure will be followed as in study 1.
- Development of the evaluation protocol:
An evaluation protocol was developed for study 2 (detailed in the variables section). Regarding the focus groups, the same procedure will be followed as in study 1.

- DBT and PU application and supervisions:
The application of the DBT and PU interventions will be in group format and will last 3 months. A group and brief format has been chosen due to the difficulties that the NHS presents in applying long-term treatments (e.g., high waiting lists, delay between sessions, etc., 82). In addition, as discussed above, the scientific literature suggests good results of these interventions using a group format with a duration of 3 months. The interventions will be adapted according to the context in which they are applied after the feedback collected in study 1, but some general aspects are detailed here.
1. DBT-SUD:
This program will consist of applying the DBT skills training mode for 3 months. We will apply the DBT-SUD (41) skills training modules: mindfulness skills, distress tolerance, addiction skills, emotional regulation and interpersonal effectiveness. Regarding the specific contents, we will rely on the program published by the team of Cesare Maffei (DBT certified trainer and therapist in Italy), which has shown good results in improving the severity and frequency of alcohol use and emotional dysregulation with a 3-month program (36).
2. PU:
The PU is made up of 8 treatment modules that include training in 5 core skills of

emotional regulation: mindfulness training, cognitive flexibility, identification/oppositional behavior of emotional behaviors, interoceptive exposure and emotional exposure (27,43). Our team has made an adaptation of the PU to be implemented in 12 group sessions, one a week, of 2 hours duration in NHS specialized mental health care services (83). In 2019, we published a book chapter entitled "Group application of the PU for the transdiagnostic treatment of TEs with comorbidity with substance use disorders" (51) resulting from the supervision carried out on a psychologist specializing in clinical psychology who worked in a UCA in Alava. This chapter describes the recommendations for the adequate implementation of the PU, module by module.

Supervisions:

The supervisions will follow the following procedure: in the first supervision session the supervisors will explain to the participants the objectives of the first therapy session, its exercises, examples and also problems that could arise and how to solve them.

In subsequent supervision sessions, the structure will be as follows: participants tell how the session went and supervisors will ask questions to assess the degree of fidelity with which the session has been followed. Based on the answers, constructive feedback will be given to the participants to reinforce the strong points and solve the weak points or deficiencies. Finally, the objectives of the second therapy session, its exercises, examples and also problems that could arise and how to solve them will be explained. This procedure will be followed in the same way for both therapies until the end of the therapeutic groups. It is expected that the interventions will be implemented over 12-14 treatment sessions so the supervision will last approximately 3 months. The Zoom platform will be used to carry out the supervisions. Here we will describes the privacy policy: https://explore.zoom.us/es/gdpr/

- Evaluation phases:

The following table summarizes the evaluation measures in each phase of study 2.

measurementsQualitative measurements	Evaluation	phaseQuantitative
Prior to implementation	- Information questionnaire of the professional<br>- Development of a standardization measure (NoMAD)<br>- Burnout Questionnaire (CBI)	
During		*implementation* Records on fidelity with the intervention
After implementation	- Acceptability (AIM), Opportunity (IAM) and Feasibility (FIM) measures of the intervention.<br>- Barriers to Implementation Inventory (BTI)<br>- Normalization MeAsure Development Questionnaire (NoMAD)<br>- Implementation	Climate Scale (ICS)<br>- Organizational disposition

- Focu s grou	p qualitative measures of implementation (acceptability,	adoption, reach, feasibility, reliability) and implementation barriers and facilitators

	for the implementation of change (ORIC)<br>- Evidence-Based Practice Attitudes Scale (EBPAS).<br>- Sustainability (PSAT)<br>- Burnout Questionnaire (CBI)<br>- Brief Understanding Substance Abuse Scale (SUSS)<br>- Acceptability and intentionto use survey (Peris-Baquero et al., 2021).	

Statistical analysis:

Study 1: Quantitative data: Data will be analyzed in SPSS V25.0 statistical software (84). First, descriptive statistical analyses will be carried out to analyze the characteristics of the participants (means and standard deviations for continuous variables and frequencies for categorical variables). Subsequently, analysis of variance (MANOVA) or Chi-square tests (depending on the variables to be analyzed) will be carried out, with the aim of exploring the existence of differences (sociodemographic and in the variables under study) between participants who receive DBT or PU training first. Similarly, these analyses will be used to explorethe changes that occur during the different evaluation moments. Finally, the analyses will include the calculation of effect sizes (Cohen's d), to assess the magnitude of change. A subgroup analysis by gender will be included in the analysis and interpretation of results. All scientific papers will take into account the gender and age of the participants. Qualitative data: The MAXQDA (85) statistical program will be used for the qualitative analysis of the information obtained through the focus groups. First, a word-by-word transcriptionof the focus groups will be made. Based on the procedure described by Schreirer (86), the transcribed information will be analyzed. This procedure makes it possible to construct a hierarchical coding system that allows the extraction of "general categories", which are composed of "subcategories" and their corresponding "areas" or "themes". In order to ensure reliability in the information extraction process, the extraction of the "categories", "subcategories" and "areas" will be carried out by two independent researchers (VM and OP). In case of disagreement, a third researcher will be consulted (researcher triangulation). In addition, the degree of agreement between the initial draft and the final version of the information extraction will be calculated through the calculation of Cohen's Kappa.

Study 2: In addition to using analyses similar to those carried out in study 1, Pearson's R correlation and multiple linear regression analyses will be included, with the aim of exploring the relationship between the different variables and identifying which variables have a predictive role in the results. These analyses will be carried out using SPSS V25.0 statistical software (84).

Limitations of the study:

The limitations or obstacles we may encounter in conducting the study are as follows:

The study will be carried out mainly in public or subsidized centers of our NHS, so the results cannot be generalized to other health contexts. Despite this limitation, we consider that the results of the project could lay the foundations of a quality procedure for the implementation of EBT for alcohol addiction, so that health managers and mental health professionals working with this population in other contexts could replicate the

proposed procedure and compare its results with those obtained by our project.

2. Failure to reach a sufficient sample to carry out the 2 studies planned. Given that participation is voluntary, it could be the case that, despite receiving the institutional email to encourage participation in the study, the necessary participation is not achieved. If this were to happen, we could invite other autonomous communities to participate in the project, given that the project represents an important economic saving for the administrations in the training of professionals. The members of the research team have contacts with the autonomous communities of Andalusia, the Basque Country and Madrid.

3. Some of the questionnaires we will use are not yet validated for use in Spanish due to the scarcity of studies of this type in Spain. This could reduce the validity of some of the conclusions obtained. However, to avoid this, the relevant translations and adaptations to be able to use the scales have been carried out following the general recommendations for this purpose (independent translation by two translators, back-translation, final version by native speakers and experts in the field). We also plan to analyze the psychometric properties of the tests used to determine whether they meet the standards of validity and reliability required for their use.

4.7 Ethical aspects (risk/benefit balance, **justification in case of requesting exemption from** informed **consent**, care implications, implications for the participant or his/her family, compensation to participants, insurance policy).

Attached is the authorization document for data processing by Unizar.
In this case, online surveys will be used. Before each survey, an information and informed consent form will be included. In case they are interested in participating, participants will accept the consent option to participate in the study, through the Qualtrics platform (see informed consent). Focus groups will also be conducted via videoconference (explained in the procedure section) through the zoom platform. Both platforms have a privacy and data protection policy: Zoom (https://explore.zoom.us/es/gdpr/), Qualtrics (https://www.qualtrics.com/support/es/survey-platform/getting-started/data-protection-privacy/). The information and informed consent sheet details all the ethical aspects related to the research.

Risks and benefits: these are as stated in the information document "Possible side effects of participation include stress or discomfort". Answering the questions in this survey may seem personal or may cause discomfort. The investigators will take care to protect confidentiality. If you begin to experience any side effects, you may stop responding at any time. As this is a knowledge-oriented research study, you are not likely to receive any benefit from your participation, although you will contribute to the scientific advancement and benefit of the study.
social. You will not receive any financial compensation for your participation."

4.8 Schedule and work plan:

- Development stages, duration, start and end dates,
- Places where the project is planned to be carried out, facilities to be used.

Stages of development, duration, start and finish dates

The following table shows the development stages with duration and dates.

Phase	Task	Task description	Start Date	Date End
Phase 0: Coordination	0.1. Literature review and task delimitation	Review the latest research results in the field of the study and set out the specific tasks for each person in charge.	01/01/2024	01/02/2024
Study 1_Phase 1. Development of the	1.1. Protocol design evaluation	Develop the protocol for evaluation of study 1.	01/02/2024	01/06/2024

evaluation and recruitment protocol	Construction of online questionnaires and database preparation	Build the evaluation surveys in Qualtrics and prepare the SPSS database.	01/02/2024	01/06/2024
Study 1_Phase 2. Dissemination of the project	2.1. Dissemination of the Study Protocol	Registration of the study and preparation of the article on the study protocol	01/06/2024	01/1/2025
Study 1_Phase 3. Therapist attitudes towards interventions	3.1. Recruitment of Professionals	Contacting professionals to ask for participation in the study 1	1/06/2024	01/02/2025
	3.2. Passing of protocol evaluation prior to the formations	Administration of the survey with questionnaires prior to training	01/02/2025	01/03/2025
Study 1_Phase 4. Training in emotional regulation interventions and assessments	4.1. Training of DBT professionals	To train professionals in Dialectical Behavioral Therapy adapted to addictions.	01/02/2025	01/03/2025
	4.2. Post-DBT evaluation protocol pass	Administering the survey after training in DBT	01/03/2025	01/04/2025
	4.3. Training of PU professionals	To train professionals in a unified protocol adapted to addictions.	01/02/2025	01/03/2025
	4.4. Post PU evaluation protocol pass	Administer the survey after PU training	01/03/2025	01/04/2025
Study 1_Phase 5. Evaluation of the acceptability and intention to use the interventions.	5.1. Conduct focus groups	Evaluation of the acceptability, intention to use and possible adaptations of the interventions for subsequent implementation in the professionals of each autonomous community.	01/04/2025	31/04/2025
Study 1_Phase 6. Statistical analysis and interpretation of results	6.1. Analysis of the results of the study and interpretation	Analyze the quantitative and qualitative measures collected in each phase.	01/03/2025	01/05/2025
Study 1_Phase 7. Dissemination of results	7.1. Dissemination of Study Results	Preparation of articles and conference presentations	01/03/2025	01/05/2025
Study 2_Phase 1. Recruitment of therapists	1.1. Recruitment of therapists	Recruitment of trained therapists who wish to implement the interventions	01/05/2025	01/06/2025
Study 2_Phase 2. Adaptation of the intervention protocol	2.1. Adaptation of the intervention protocol	Adaptations of DBT and PU intervention protocols according to the context in which they are implemented.	01/06/2025	01/07/2025
Study 2_Phase 3. Pre-implementation protocol pass	3.1. Evaluation of the adequacy of interventions	Administer the survey prior to implementation to assess the adequacy of the interventions	01/09/2025	31/09/2025
Study 2_Phase 4. Implementation of interventions and supervision of therapists	4.1. Supervision of therapists	Weekly monitoring sessions during implementation to assess fidelity of intervention delivery	01/09/2025	01/12/2025
Study 2_Phase 5. Evaluation of the implementation process	5.1. Evaluation of implementation process measures	Evaluation of implementation measures: acceptability, adoption, scope, feasibility, fidelity during and after implementation	01/12/2025	31/12/2025

	5.2 Focus groups: evaluation of the implementation process	Qualitative evaluation: implementation process and post-implementation barriers and facilitators.	01/12/2025	31/12/2025
Study 2_Phase 6. Statistical analysis and interpretation of results	6.1. Analyze the results of the study and interpret them	Analyze the quantitative and qualitative measures collected in each phase.	01/9/2025	31/12/2025
Study 2_Phase 7. Dissemination of results	7.1. Dissemination of Study Results	Preparation of articles and conference presentations	31/12/2025	An extension of the study will be asked to conduct the dissemination

Project sites and facilities:

The project management activities will be carried out by the members of the research team at the Faculty of Social and Human Sciences of the Teruel Campus of the University of Zaragoza. DBT and PU training will be conducted in online format. The administration of questionnaires will be done through the Qualtrics platform. Focus groups and supervisions will be carried out through the Zoom platform.

The members of the research team belonging to the Faculty of Social and Human Sciences of the University of Zaragoza (Teruel Campus) have offices for teaching and research use. Therefore, the activities and tasks necessary to carry out the study can be carried out without additional costs. In the campus we have 10 computers, 2 of them laptops for the Basic and PETRA areas, 2 printers (one laser). The computers will be used to send emails, do bibliographic searches, write research papers, create the database, enter data and perform statistical analysis in SPSS, write the report, etc. and the printers will be used to print the necessary documents. They may also use the telephone for coordination of the research team. In the case of the collaborating personnel, they also have their own office with resources that will allow them to carry out the research tasks at no cost. The instruments included in the project are freely available for the research, so there is no added cost. Some expenses are added in the budget to have additional material means to carry out the project.
